# Emergent dynamics in a model of visual cortex

**DOI:** 10.1007/s10827-013-0445-9

**Published:** 2013-03-22

**Authors:** Aaditya V. Rangan, Lai-Sang Young

**Affiliations:** Courant Institute of Mathematical Sciences, New York University, New York, NY USA

**Keywords:** Visual cortex, Cascade, Dynamical systems

## Abstract

This paper proposes that the network dynamics of the mammalian visual cortex are highly structured and strongly shaped by temporally localized barrages of excitatory and inhibitory firing we call ‘multiple-firing events’ (MFEs). Our proposal is based on careful study of a network of spiking neurons built to reflect the coarse physiology of a small patch of layer 2/3 of V1. When appropriately benchmarked this network is capable of reproducing the qualitative features of a range of phenomena observed in the real visual cortex, including spontaneous background patterns, orientation-specific responses, surround suppression and gamma-band oscillations. Detailed investigation into the relevant regimes reveals causal relationships among dynamical events driven by a strong competition between the excitatory and inhibitory populations. It suggests that along with firing rates, MFE characteristics can be a powerful signature of a regime. Testable predictions based on model observations and dynamical analysis are proposed.

**Electronic supplementary material:**

The online version of this article (doi:10.1007/s10827-013-0445-9) contains supplementary material, which is available to authorized users.

## Author summary

We present a parsimoniously designed model of the mammalian primary visual cortex which has been well benchmarked against experimental data, and which can capture many of the experimentally observed V1 phenomena seen in cat and monkey. During our benchmarking process, we find that the regimes which support the most biologically realistic behavior are those that exhibit temporally localized barrages of excitatory and inhibitory firing. These multiple-firing events are an important feature of the neuronal network dynamics and play a commanding role in the network’s transient and steady-state response properties. We predict that similar multiple-firing events should be observable in the real cortex.

## Introduction

The mammalian primary visual cortex (V1) plays an integral role in many discrimination, recognition and classification tasks. It performs diverse functions, and is well known to be immensely complex. As a dynamical system, V1 likely demands many degrees of freedom to support the great variety of dynamical processes accompanying its many visual tasks. The aim of the present study is to gain insight into these processes via computational modeling: We designed a mechanistic model, carefully benchmarked it using physiological data, and analyzed the underlying dynamics. Our primary finding is that, when appropriately calibrated, neurons in our network fire neither synchronously nor independently, but in a highly structured way. We propose that this is indicative of how the real cortex operates.

We elaborate on each of the main steps:

When designing our model, the challenge was to strike a balance between biological plausibility and parsimony. We have elected to use a spiking network model which we have built from the ground up, modeling its architecture on basic V1 physiology. At the same time, we have had to strip away many layers of complexity and introduce some amount of idealization, without which the analysis of dynamical mechanisms would not be possible.

The benchmarking process consisted of the following: Our network has ∼ 10 parameters (representing e.g. coupling strengths). A roughly comparable number of experimental results and known biological facts from multiple sources were used: a few were used to constrain parameters, the rest as validation. We have found that there are regions in parameter space on which the regime exhibits simultaneously all of the phenomena considered. These phenomena include firing rates, background patterns, surround suppression, gamma oscillations etc.

Our primary finding is that in the biologically plausible regimes of our network, there is strong self-organized collaborative firing activity in the form of ‘multiple firing events’ (MFEs); i.e., spiking in local populations occurs in brief ‘spurts’ of variable sizes; the time intervals between consecutive spurts are random but have characteristic lengths. These events are an emergent phenomenon, due not to correlated feedforward inputs but to keen competition between excitatory and inhibitory populations. Given that MFEs are ubiquitous in our network, and there is experimental evidence pointing to the occurrence of such events in real cortex (Samonds et al. 2005; Mazzoni et al. 2007; Petermann 2009; Churchland et al. 2010; Yu and Ferster 2010; Yu et al. 2011; Plenz et al. 2011; Shew et al. 2011), we propose that this *is* the operating point of cortical dynamics, and include some testable predictions. We know from our analysis that MFEs affect subsequent dynamics; we conjecture that they may encode useful information.

The main thrusts of this paper can be summarized as follows: We present strong evidence in support of the hypothesis that cortical activity is highly structured though irregular, due in part to the mechanism of MFEs. Our investigation, which is data-driven, has led to results which challenge prevailing views in theoretical neuroscience: Instead of focusing on (i) synchronous behavior or (ii) coarse-grained models which presume weak correlations between neurons, our results suggest that V1 is likely to operate somewhere in-between, incorporating in a complicated yet systematic way some characteristics of both (i) and (ii).

## Results

### Model design

Existing models range from very small to very detailed. The former have the virtue of simplicity but tend to be limited in scope, as they lack the structural components required to capture multiple phenomena, while the latter tend to involve a large number of parameters which cannot be appropriately constrained.

To develop a coherent and multi-faceted picture of the dynamics of V1, we have constructed a model equipped with the minimum number of architectural features necessary to support the broad range of V1 phenomena discussed below, while at the same time having few enough parameters to allow for serious benchmarking and subsequent investigation.

**Fig. 1:**
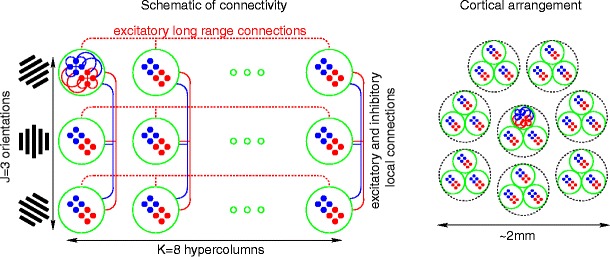
*Left:* Schematic diagram of network architecture. Our network comprises a 2D array of ‘clusters’ (*green circles*) each containing a few hundred excitatory (*red*) and inhibitory (*blue*) neurons. Rows correspond to clusters with the same orientation preference and columns represent hypercolums. We refer to *C*_*jk*_ as the *j*th orientation domain in the *k*th hypercolumn. Neurons within each cluster are connected via local excitatory and inhibitory connections (as indicated by *red* and *blue lines* within one representative cluster). Clusters with different orientation preferences within a hypercolumn (i.e., *C*_1*k*_, *C*_2*k*_, …) are also connected via local connections. Clusters of the same orientation preference in different hypercolumns (i.e,. *C*_*j*1_, *C*_*j*2_, …) are connected via excitatory long-range connections (as indicated by *dashed red lines*). Local connectivity both within each cluster and between clusters in the same hypercolumn is statistically homogeneous; neurons are sparsely and randomly connected to one another, with slightly higher connectivity among neurons within a cluster. Long-range connectivity between clusters with like orientation-preference in different hypercolumns is also statistically homogeneous. For simulations we use a network with 3 orientation domains in each of 8 hypercolumns. *Right:* Hypercolumn arrangement. Eight hypercolumns, each containing 3 clusters corresponding to distinct orientation domains, are depicted by *dashed circles*; all pairs of hypercolumns are thought of as ‘adjacent’, or ‘equal distance’ apart. Our network is intended to model a ∼ 2 mm^2^ patch of cortex: large enough to contain several hypercolumns, but small enough that the long-range connectivities between any two pairs of hypercolumns are roughly similar

The model discussed in this paper is that of a ∼ 2 mm^2^ patch of layer 2/3 of V1. We have built it out of a network of several thousand spiking integrate-and-fire point neurons, using the voltages and conductances of individual neurons as microscopic variables. To allow for basic V1 features such as orientation selectivity and receptive field structure (Hirsch 2003), we have partitioned our network into groups of neurons representing hypercolumns, each one of which is further partitioned into orientation domains. In terms of connectivity, we have abstracted the complex topology of cortical interaction as follows: Orientation domains within each hypercolumn interact via local connections, whereas orientation domains in different hypercolumns interact via orientation-specific long-range ‘horizontal’ connections. Schematic diagrams of the network architecture of our model are shown in Fig. 1.

With regard to network dynamics, we distinguish between fast and slow synaptic currents, with fast currents corresponding to AMPA-type excitation and GABA-A type inhibition and slow currents representing NMDA-type excitation with decay time $\tau _{\mathrm {slow}} \sim 100$ ms. All cells in each orientation domain are driven by independent feedforward Poisson input comprising (i) a background drive which has uniform rate across all clusters and, where applicable, (ii) a drive modeling a drifting-grating stimulus which targets stimulus specific orientation domains and hypercolumns.

These are the main ingredients in our model; further details are given in the Section 5 and in Supplementary Material. As is evident from the description above, our model is orders of magnitude simpler than the real V1. We have included only a dozen or so of the most important parameters, such as short- and long-range coupling strengths ($S^{Q'Q}$ and $L^{QE}$ respectively, for $Q,Q'=E,I$) between neuronal populations, and strengths of feedforward input currents. These parameters will be constrained by comparing network outputs with data from experiments; this is discussed in Section 3.2.

We stress that our results are *data-driven*. To ensure that we did not limit *a priori* the type of dynamics that may occur, we elected not to use from the outset coarse-grained variables such as firing rates. Had we done that, the phenomena discussed in Section 3.3 would have been missed entirely.

### Benchmarking and validation

We subjected our network to a battery of ‘tests’ in which we tried to replicate certain empirical observations of V1, and found that *there is a reasonably-sized region in parameter space on which the dynamical regime corresponding to each parameter point exhibits simultaneously all of the properties below.* Specifically, (1)–(4) below were used to constrain parameters; no further constraining was necessary for the remaining phenomena.

**Fig. 2:**
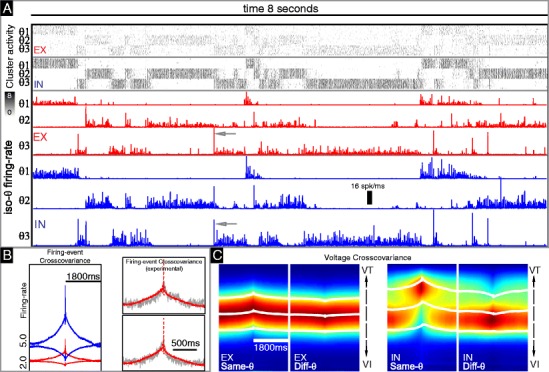
Dynamical regime exhibiting correlated background activity. The top of (**a**) shows raster-plots of the activity during an 8 second stretch of time. The clusters are organized such that the first eight clusters correspond to *θ*_1_, the second eight to *θ*_2_, and the third eight *θ*_3_. Thus, the clusters in the same hypercolumn are not plotted adjacent to one another. Each row represents the total number of firing-events of either the E-(*top*) or I-(*bottom*) population within each cluster binned over intervals of 1 ms. This total number of firing-events is represented logarithmically using the colorscale to the *left* (i.e., the value 3 corresponds to 2^3^ = 8 total events within the *N*_*Q*_ = 128 neuron population over a 1 ms interval, or an instantaneous population firing-rate of 64 Hz). In this panel it can clearly be seen that a typical epoch within this background regime is dominated by the activity of E- and I-cells associated with one of the 3 orientations. Below the rasters in panel-(**a**) we display the summed E-(*red*) and I-(*blue*) activity for each orientation. The *vertical scale-bar* represents a magnitude of 256 total events per 1 ms bin across all 8 clusters corresponding to a given *θ*—i.e., a single-cluster event-count of 16 spikes per ms. Note that when any one *θ*_*j*_ is active, the other two orientations are not typically active. Nevertheless, the activity is not always restricted to a single orientation—it is not rare for multiple orientations to activate simultaneously. Note also that, within this regime, there are multiple events which involve brief heightened activity across both the E- and I-populations in many iso-orientation clusters (see *arrowheads*). On the left of panel-(**b**) we plot the crosscovariance *C* (*E*, *Q*, *A*, 0, *τ*) in spiking activity between cells in different hypercolumns. See Section 5 for a definition of crosscovariance. The crosscovariance *C* is plotted for *Q* = *E* (*red*) and *Q* = *I* (*blue*), as well as for *A* = 1 (*upper curves*) and *A* = 0 (*lower curves*). Note that iso-orientation activity is correlated on a timescale of ∼ 300 ms, whereas non-iso-orientation activity is anticorrelated over a similar timescale. Also note that the crosscovariance is nearly symmetric and positive for both *Q* = *E* and *Q* = *I*. In the right of panel-(**b**) we show experimental crosscovariance measurements from two paired recordings presented in (Kohn and Smith 2005) (*gray*, reproduced with permission), superimposed with the crosscovariance *C* (*E*, *E*, 1, 0, *τ* measured in our simulation (*light red*, taken from top of panel-(**b**)). Although the vertical scale is not directly comparable, the persistence time-scale observed in experiment is similar to that seen in simulation. In panel (**c**) we plot the spike-triggered average voltage-distribution (see Section 5) *SVD*(*E*, *Q*, *A*, 0, *τ*, *V*) for *Q* = *E* and *Q* = *I* and *A* = 1 (Same-*θ*) and *A* =  0 (Diff-*θ*). The *solid white lines* indicate the mean and mean±stdev for the voltage distribution

1. *Firing rates and EPSPs:* In real V1, E- and I-firing rates in background (i.e. when unstimulated) are known, as are sizes of EPSPs. We require that corresponding values for our network lie within acceptable ranges.
2. *Receptive fields and orientation tuning:* (i) The idea of classical receptive fields in V1 is translated in our network to requiring that cells in *C*_*jk*_ not be activated by stimuli applied to *C*_*jk*′_ for $k' \ne k$ (see caption of Fig. 1 for notation). (ii) We require that E-neurons be sharply tuned for orientation, i.e., firing in *C*_*jk*_ should be lower when $C_{j'k}, j' \ne j$, is stimulated than when no domain is stimulated; we also require that I-neurons be broadly tuned. We demonstrate also ‘sharp tuning’ using a version of our model with $J=4$, showing that such a model produces sharply tuned firing-rates even when driven by weakly tuned feedforward input. See Section 3.3 in Supplementary Material for more details.
3. *Iso-oriented surround suppression:* Here we view a stimulus applied to a single cluster *C*_*jk*_ as representing a small drifting-grating stimulus aligned with orientation *j*, and a stimulus applied to $\cup _{k'} C_{jk'}$ as one simulating a full-field drifting-grating. We require the following: (i) When the system is strongly driven by an input targeting cluster *C*_*jk*_ alone, the steady-state firing rates of both E- and I-populations in *C*_*jk*_ should increase significantly from their background values; (ii) when the input is expanded to target $\cup _{k'} C_{jk'}$, firing rate of the E-population should drop significantly from that in (i). This is consistent with experimental results in real V1 (Sceniak et al. 1999). As to the I-population, there has been an assumption in the community that (iii) I-firing rate increases as we go from the stimulus in (i) to that in (ii), while recent experimental results of (Ozeki et al. 2009) report (iii’) a decrease in the corresponding firing rate. We have located two (distinct) parameter regions, one exhibiting (iii) and the other (iii’), along with all the other properties listed; the region corresponding to (iii) is considerably larger. Dynamical mechanisms leading to the two different outcomes are discussed in Section 3.4. See (9) below for a related result.
4. *Spontaneous correlated background activity:* Unstimulated background activity is observed in the visual cortex of several mammals (e.g., ferret, cat, and monkey). One sees spatio-temporal structure with spatial correlations across orientation domains of similar orientation preference in nearby hypercolumns and temporal correlations on the order of 50–500 ms (Tsodyks et al. 1999; Kenet et al. 2003). In our model we coarsely discretize orientation, so the orientations represented by different clusters in a hypercolumn are quite dissimilar. Thus, as acceptable background activity in our network, we require sustained activity patterns that correspond to increases in both mean firing-rate and mean subthreshold voltage across all the clusters of a given orientation *θ*; clusters with a different *θ* should not be concurrently activated during much of this period though some concurrent patterns are permitted from time to time. Furthermore, we require that any given sustained activity pattern should persist for $\tau _{\text {persist}}^{\text {bkgrnd}} $ ∼50–500 ms before either decaying to a ‘*θ*-mixed’ state or evolving into another sustained pattern associated with a different *θ*. Some of our findings are illustrated in Fig. 2.

We emphasize that, in accordance with (Chiu and Weliky 2001), the correlated activity observed in background is entirely emergent, and is not dependent on structured input (the feedforward input to the neurons in our model is pure Poissonian, and is independent across neurons). Moreover, the durations of the patterns are quite random and often ill-defined, but they have a characteristic persistent time $\tau _{\text {bkgrnd}}^{\text {persist}} $ which can be considerably longer than *τ*_slow_; this is consistent with several experimental observations (Tsodyks et al. 1999; Chiu and Weliky 2001; Kenet et al. 2003).

The parameters selected to fit (1)–(4) above also satisfy (5)–(9) below. This can be taken as validation of our model and parameter choices.

**Fig. 3:**
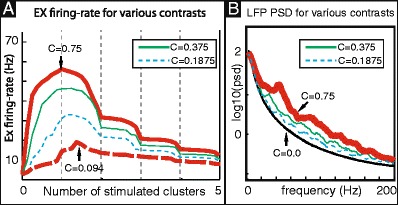
**a** Here we show the time-averaged excitatory firing-rate as a function of stimulus size (i.e., the number of driven clusters) across a variety of contrasts. Each curve indicates the firing-rates corresponding to a different fixed contrast. The contrasts chosen for illustration are $C=C_{E,j,k}=0.7500,0.3750,0.1875,0.0938$ (see Section 5), with the highest and lowest contrasts indicated by *red solid* and *dashed lines*, respectively. Note that, for low contrast, the firing rate does not decrease appreciably when the size of the stimulus increases from 1-cluster to 2-clusters, whereas for high contrast, the corresponding decrease is significant. **b** Power spectral density (averaged over nonoverlapping 512 ms windows) of the population averaged voltage for a single cluster in our model both under background (*black*) and under low-contrast (*green*) and high-contrast drive (*red*)

(5) *Contrast dependence:* Using Poisson rates in our external input as an indication of contrast, and the number of clusters (of the same orientation) stimulated as representing the diameter of the drifting grating, we find the response in terms of excitatory firing to be consistent with those in (Sceniak et al. 1999), i.e. maximum firing occurs at larger stimulus size for weaker contrast. See Fig. 3a.
(6) *Gamma-oscillations in LFP:* When averaged over the neurons in a cluster, the subthreshold voltage is highly irregular, exhibiting broadband fluctuations. Moreover, the power spectral density of this locally averaged voltage signal shows an increase across the gamma-band when the system is driven, consistent with LFP recordings of V1 (Henrie and Shapley 2005). See Fig. 3b.
(7) *Variance reduction at onset of input:* Following stimulus onset, the trial-to-trial coefficient of variation of individual neuronal responses drops considerably, while still maintaining relatively high values. This is consistent with measurements of V1 (Churchland et al. 2010).
(8) *Shift in coherence at onset of input:* Following stimulus onset the high frequency components of the population’s activity become more coherent, whereas the lower frequency components become less coherent (see, e.g., Fig. 5c and e). This is consistent with measurements of V1 (Yu and Ferster 2010).
(9) *Surround suppression for natural stimuli.* When the drifting grating stimulus used in (3) above is replaced with a more natural stimulus with rich spatio-temporal structure, it is reported that I-firing increases when stimulus size is increased (Haider et al. 2010). We have produced similar results for certain non-iso-oriented stimuli.

We were far from the first to attempt to model V1 phenomena. Earlier works include (i) the firing-rate models of (Murphy and Miller 2009) which exhibit orientation tuning and a form of background fluctuations dependent on correlated input, (ii) the firing-rate models of (Ozeki et al. 2009) which exhibit surround suppression of the inhibitory population, (iii) spiking network models of (Battaglia and Hansel 2011) which exhibit orientation tuning and broadband gamma-band oscillations, and (iv) spiking network models of (Rangan et al. 2005) which exhibit spontaneous background patterns and dynamic transients associated with the Hikosaka line-motion-illusion.

We reiterate that our model exhibits simultaneously, in a single regime, all of phenomena (1)–(9). Proposed explanations for some these phenomena are given in Section 3.4. See also Supplementary Material.

### MFEs: an emergent phenomenon

On large regions of parameter space—considerably larger than the physiologically plausible region found in Section 3.2—neurons in our network spike neither synchronously nor completely independently; the network activity is somewhere in between and highly structured. The most readily apparent of these structures are sudden barrages of excitatory firing that are temporally localized, accompanied by a commensurate amount of inhibitory firing. These barrages typically involve, but are not limited to, single clusters (see Fig. 1). We call them ‘*multiple-firing events*’ (MFEs).

Now we do not claim that this phenomenon is entirely novel: It is known that neurons in real V1 can fire in partially synchronous bursts (Samonds et al. 2005; Mazzoni et al. 2007; Petermann et al. 2009; Churchland et al. 2010; Yu and Ferster 2010); it has also been noted that spiking network models can have MFE-like behavior (Hansel and Sompolinsky 1996; Brunel 2000; Kriener et al. 2008; Poil et al. 2012). Specifically, our MFEs are almost for certain related to neuronal avalanches, which are well documented in both models and experiments (Yu et al. 2011; Plenz et al. 2011; Shew et al. 2011). Leaving comparisons for later, we first give a mechanistic description of how MFEs are produced.

#### Dynamics of MFEs in single clusters

We consider here a single ‘cluster’ as defined in Fig. 1, consisting of an E- and an I-population both driven by Poissonian inputs, and discuss in this simpler setting the dynamics of MFEs: how they are generated, their aftermath, and associated timescales. In our full model (as described in Section 3.1), MFEs are produced similarly, except that inter-cluster competition further complicates the picture.

**Fig. 4:**
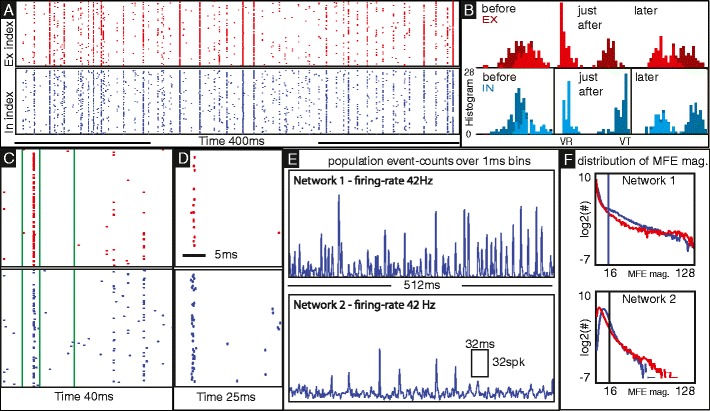
A pictorial introduction to MFEs. **a** Raster plots for E (*top*) and I (*bottom*) cells in a single cluster of size $N_E=N_I=128$. The dynamic regime is chosen to clearly exhibit MFEs. *Near-solid vertical lines* correspond to large MFEs; notice that MFEs in the E-population elicit similar-sized MFEs in the I-population. Notice also the characteristic (but variable) time intervals separating consecutive MFEs. **b** Voltage distribution for the E (*top*) and I (*bottom*) cells in the cluster both before (*left*), shortly after (middle) and ∼ 10 ms after (*right*) the first large MFE shown in panel-(**c**). The times shown are indicated by green lines in panel-(**c**). The brighter population (*vermillion, cyan*) corresponds to the fraction of E and I cells which fire during the MFE. Note that, during the MFE, the firing E-population crosses threshold and returns to reset, whereas the nonfiring population is suppressed. After the MFE has concluded, the entire population slowly drifts closer towards the pre-MFE distribution. This is a very large MFE, selected for easy visualization. Smaller MFEs involve the crossing of threshold by a few neurons to a smaller fraction of the population, but the mechanism is the same. **c** Zoom in on a few MFEs produced by the cluster in panel-(**a**). **d** A raster showing an MFE in a larger cluster of conductance-based integrate-and-fire neurons with fast-conductance timescales ∼ 2 ms and $N_{E}=N_{I}=2048$. We show this to demonstrate the following two facts: (i) The phenomenon of MFEs persists when we let fast-conductance timescales take on more realistic values. We set them to 0 in the text in order to make precise the definition of an MFE, but that is not necessary, because a few ms is still considerably smaller than the membrane time constant *τ*_*V*_ = 20. (ii) MFEs are not a finite-size effect; they can be shown to occur in arbitrarily large clusters provided parameters are tuned appropriately. **e** Population spike-counts for two single-cluster networks with the same firing-rate, but with very different MFE signatures, one producing frequent large MFEs and the other producing a more steady stream of independent firing events. If these characteristics affect computation downstream, then firing rate alone would not adequately describe a regime. **f** We plot, on a log-scale, the distribution of spike-counts for the two networks shown in panel-(**e**). The vertical line at 16 spikes/bin indicates the cutoff for the ‘95 % rule’ described in Section 3.4. These plots show that the distribution of MFE magnitudes that appear is very rich; they vary widely from very small to very large. Notice also that the frequency of MFEs and the distribution of MFE magnitudes is very different for these two regimes, an observation which will be overlooked by trial-averaged or time-averaged firing-rates

To understand how MFEs come about, we assume, to begin with, that the fast synaptic currents have zero rise and decay times (this idealization can be removed; see Fig. 4d and Supplementary Material). Suppose at some moment an E-neuron fires. This increases the voltages of a number of E- and I-neurons, and if some of these voltages are sufficiently close to threshold, the initial spike can ‘cause’ some postsynaptic neurons to fire. Suppose, by chance, the first E-spike causes some other E-cells to fire. That in turn raises the voltages of more cells, possibly setting off a chain reaction until eventually enough I-cells are aroused, and their inhibitory action terminates the barrage of firing. This is what we mean by a ‘multiple firing event’ (MFE). See Fig. 4b,c for illustration.

While the propensity of a regime to nucleate MFEs is a function of the synaptic coupling strengths $S^{Q'Q}, Q, Q'=E,I$ and background drive (see below), the exact details of what happens in the 0–5 ms following the initial E-spike depends sensitively on the voltage-configuration of the system just prior to the spike: whether the next few neurons to cross threshold are excitatory or inhibitory will determine if an MFE will follow. The ‘magnitude’ of the MFE, i.e., the total number of E- and I-cells which fire during this brief transient, can be any number > 1 to the entire cluster, though frequent large MFEs—i.e., near-synchronous firings—are likely unbiological (and are not permitted in admissible regimes in Section 3.2).

Immediately following an MFE, the system is in a rather different state: neurons that participated in the MFE are in or just coming out of their refractory periods, while the voltages of much of the rest of the population are compacted somewhat and set far below threshold due to the final surge of I-synaptic current which concluded the MFE (as I-neurons arborize fairly densely). See Fig. 4b. In a nontrivial-sized MFE, this current pushes the E-population sufficiently far back that there tends to be a 10–25 ms lull in activity (the length of the lull depending on input drive) before the compacted population is ‘recharged’, and has the capability to nucleate another MFE.

We emphasize that the MFEs in our system are not due to correlations in the feedforward input (which are uncorrelated across neurons), nor are they due to synfire pathways embedded in the local connectivity (which is random and statistically homogeneous). While the addition of these features would increase the likelihood of generating MFEs, they are certainly not required. Rather, MFEs in our system are generated internally as a result of the dynamics, i.e., this is an *emergent* phenomenon.

As to which conditions contribute to the precipitation of MFEs, a rule of thumb is the following: If synaptic couplings and background drive are balanced in such a way that either (i) the I-population dominates, or (ii) both distributions are far from threshold, then firing events are likely to involve single neurons. If the E-population has too much of an advantage, then near-synchronous firing will result. In situations where there is genuine competition between the E- and I-populations, MFEs will likely ensue, due to normal fluctuations magnified by the tendency for E-firing to promote further spiking. They are prevalent in regimes in which a small perturbation or fluctuation can have a large effect, as is consistent with what is known about V1 (Lampl et al. 1999; Anderson et al. 2000).

**Fig. 5:**
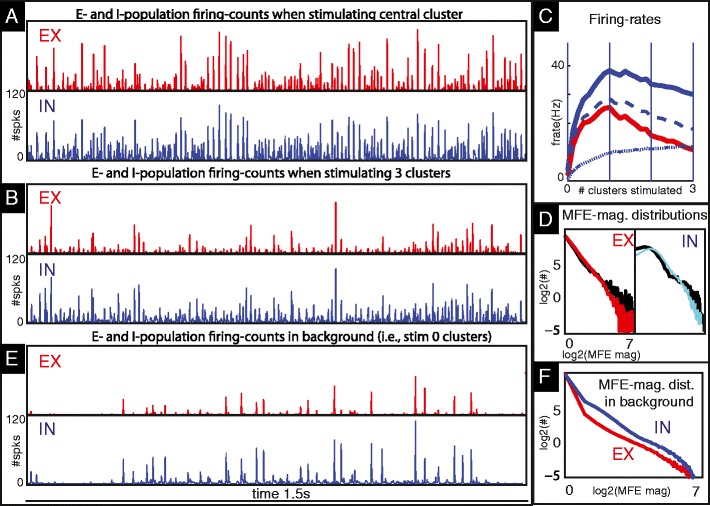
In panels (**a**) and (**b**) we show the firing-events produced by the ‘reference parameter set’ used in Fig. 2 under drive. **a** illustrates the sequence of E- and I-population spike counts (collected over 1 ms bins) produced by the central cluster *C*_*ij*_ in our network when that cluster alone is stimulated. **b** illustrates these spike-counts when 2 other clusters *C*_*jk*′_ of similar orientation preference in other hypercolumns are also stimulated. In (**c**) we show time-averaged firing-rates for the E-(*red*) and I-(*blue*) populations as a function of the number of clusters stimulated. The firing-rates for the I-populations are further divided into *MFE-spike* (*dashed*) and *nonMFE-spike* (*dotted*) rates, as per the ‘95 %’-rule described in Section 3.4. Note that, as the number of stimulated clusters increases, the independent I-firings increase a little, but the MFE-triggered I-firings decrease in frequency noticeably, and as a result the total I-firing-rate decreases as the stimulus size increases. In (**d**) we show the histograms of MFE magnitudes for E-(*left*) and I-cells (*right*) for the regimes shown in panels (**a**) (*black*) and (**b**) (*colored*) respectively. Note that for the large stimulus used in panel-(**b**) (*colored*) there are fewer large MFEs and more small MFEs than when only a single cluster is stimulated (*black*). In (**e**) we show for reference the population spike counts for the central cluster during a typical active epoch in background (taken from Fig. 2). In (**f**) we show the MFE distributions for the E-(*red*) and I-(*blue*) cells in background (collected over a much longer time than that shown in panel-(**e**)). These MFE-distributions generated by our network are qualitatively similar to the burst distributions observed in (Mazzoni et al. 2007). Note that, under drive, the low-frequency background fluctuations are suppressed, and the higher-frequency sequences of MFEs become more tightly packed (compare panel-(**e**) with panel-(**a,b**)). This contributes to the stimulus-driven coherence shift mentioned in (8) of Section 3.2

Between completely uncorrelated activity and total synchrony lies a vast and varied dynamical landscape, a more detailed analysis of which is given in (Rangan and Young 2012). An example of the mixed behavior typically seen in our network is shown in Fig. 5a and b.

### MFEs in V1 dynamics

We now return to our network model of V1. All of the physiologically plausible dynamical regimes we have found exhibit nontrivial MFEs, both in background and under drive. The MFEs seen often result from interactions of the E- and I-populations within a cluster, modulated by inter-cluster interactions. We discuss some examples of this mechanism at work:

Items (6)–(8) in Section 3.2 are fairly direct consequences of the presence of MFEs. Specifically, the ‘stochastic rhythm’ produced by MFEs in our driven network is largely responsible for the *gamma oscillations* in the power-spectrum of its ‘LFP’. More precisely, during most MFEs, the net synaptic influence of the firing population significantly reduces the subthreshold voltage of the nonfiring-population. Inter-MFE intervals, which include recharge times for the depressed voltages followed by a (random) waiting time for the next MFE, correspond to frequencies in the gamma-range. Times between successive MFEs are quite variable depending on the size of the MFE and the recent history of the network; this is reflected in the broadband signature observed in the power spectrum. *Coherence shift* is explained by the greater regularity of MFE occurrence when a system is stimulated, together with the fact that MFE recharge times decrease as stimulus strength is increased. Variance reduction is explained similarly.

#### Iso-oriented surround suppression

First we explain the decreased E-firing. When a cluster *C*_*jk*_ (see Fig. 1) alone is strongly stimulated, it produces fairly large MFEs. When $\cup _{k'} C_{jk'}$ is stimulated, long-range effects from $C_{jk'}, k' \ne k$, are felt by neurons in *C*_*jk*_. Since these effects are, on balance, suppressive (reflecting the fact that horizontal excitation affects I-neurons more than E-neurons), MFEs in the E-population are reduced in size and frequency leading to lowered E-firing rates.

As discussed earlier, I-firing can go up or down depending on parameter region. We have found that which way it goes is closely related to the *homogeneity* of population spike patterns. One way to capture this idea is to distinguish between ‘MFE-spikes’ and ‘nonMFE-spikes’. The following is an operational definition: Consider timebins of 1–5 ms (binsize should be comparable to or larger than the fast conductance timescales but substantially smaller than the recharge time between MFEs). Given a dynamic regime, if one assumes (i) all neurons fired independently and (ii) all spikes were equally likely to occur at all times, then the distribution of spikes in an arbitrary timebin would be a binomial distribution the mean of which is determined by firing rate and population size. For simplicity, let us agree to use the following ‘95 % rule’, which labels a timebin as an ‘MFE-bin’ if it contains more spikes than the 95th percentile in the binomial distribution. All spikes in ‘MFE-bins’ are then called ‘MFE-spikes’, and others are ‘nonMFE-spikes’. The fraction of spikes that are MFE-spikes can then serve as a measure of the homogeneity of the population spike pattern.

We are now ready to discuss the changes in I-firing at the center when $\cup _{k'} C_{jk'}$ is stimulated. When E-firing goes down, the number of MFE-spikes in the I-population goes down with it, as these spikes are largely in response to MFEs in the local E-population. However, the number of nonMFE-I-spikes at the center can go up, due to the added long-range excitation received by the E- and especially the I-populations. At issue is the *fraction* of MFE-spikes among all I-spikes in *C*_*jk*_ when only *C*_*jk*_ is stimulated. If this fraction is large, then decreasing it will lead to a decrease in the I-firing rate. If it is small, then the decrease in MFE-spikes can be compensated for by the increase in nonMFE-spikes, leading to an increase in the overall I-firing rate. This explanation is consistent with simulations. The case where I-firing decreases is illustrated in Fig. 5.

#### Background patterns

This is the most dynamically complex of the phenomena discussed, and a more in-depth analysis is given elsewhere. Here we mention only how MFEs assist in the switching of patterns: In our model, this phenomenon has much to do with the competition between the different orientation domains within a hypercolumn. While a switch can occur due to chance, the ‘time gaps’ between MFEs in the activated domain provide natural openings for attempted take-overs by a different *θ*. Thus the switching mechanism in our model is different from that in (Murphy and Miller 2009), which uses correlated input, or in (Goldberg et al. 2004), which relies solely on large-deviations in the input.

To demonstrate that the occurrence of MFEs and background fluctuations in our regime are robust and are not sensitive to certain model details, we have performed simulations to confirm the following: MFEs are clearly visible with $\tau _{\text {fast}}^{E,I}$ ∼ a few ms (they are taken to zero in the model); increasing synaptic delays in horizontal connections to ∼ 10 ms has no appreciable effect on the dynamics, and background patterns persist even as the number of neurons in each cluster *N*_*Q*_ is increased; see Sections 2.1, 2.2 and 3.3 in Supplementary Material. We have also investigated versions of our model with 4–6 orientation domains, and believe that our main results are reproducible after retuning of parameters.

## Discussion

To summarize, we have produced a network model of V1 with ∼ 10 parameters, and have benchmarked it with ∼ 10 empirically observed phenomena. Our results are data driven, and in the biologically plausible network regimes that we have found, we have identified MFEs as possibly the single most important dynamic mechanism. Using these ideas, we have proposed explanations for some of the phenomena observed, explanations that are confirmed by simulations.

### Model predictions

Given (i) the prevalence and importance of MFEs in our model, and (ii) the ample evidence that points to large fluctuations in real V1 (Samonds et al. 2005; Mazzoni et al. 2007; Petermann et al. 2009; Churchland et al. 2010; Yu and Ferster 2010; Yu et al. 2011; Plenz et al. 2011; Shew et al. 2011), we hypothesize that V1 is capable of producing MFEs. *A primary prediction that results from this work is the presence of abundant non-negligible MFEs within layer 2/3 of V1.* The occurrence of MFEs may depend on a variety of factors such as whether or not the subject is awake and behaving, details of the experimental preparation (e.g., the choice of anaesthesia), the type of stimulus used, as well as the degree to which inputs are correlated, but we expect that MFEs will be found, and propose that their existence be directly confirmed using a sufficiently dense multiple electrode array. Specifically:

- While the MFEs in our model are idealized, we expect that the MFEs in the real V1 will be quite similar to those we describe. We propose that, within local regions in layer 2/3 of V1 spanning 100–200 *μ*m, brief transient surges in neuronal activity involving collections of neurons will occur with notable frequency. These brief surges—incorporating both excitatory and inhibitory neurons—will initiate and subside on a timescale roughly equivalent to the synaptic time-constants of the system (i.e., within, say, 3–9 ms). Moreover, we expect that these events will significantly impact subsequent dynamics in the local regions in which they occur; in particular ‘inter-MFE’ intervals should not be exponentially distributed.
- We propose that the presence and characteristics of MFEs may be used as a signature of a regime, along with standard measures such as firing rates, correlations between pairs of neurons, LFPs etc. MFE characteristics can be useful where (i) the activity of the population as a whole matters more than the behavior of each individual neuron, and (ii) transient fluctuations and impulse-response properties of V1 over shorter (e.g. < 10 ms) time windows affect significantly the computation downstream. Averaging experimental observations over multiple trials or over time windows that are too long may hide the rich structure produced by MFEs on finer temporal scales. Even time-averaged correlations between pairs of neurons (which sometimes participate in these events together and sometimes not) may not reveal MFE information. There are many ways to detect or quantify the presence of MFEs. Some examples were discussed in this paper: distributions of MFE magnitudes, measures of homogeneity in population firing, e.g. the ‘95 % rule’ and other variants thereof.
- Another prediction is that MFE characteristics in the real V1, such as the shape of the distribution of population spike-counts, should (i) depend on the stimulus and (ii) influence the dynamics, as they do in our model. Specifically, under high contrast stimulus the ratio of large spike-counts to small spike-counts (over small time-windows) should decrease as stimulus size is increased.
- Last but not least, as we have explained in Section 3.3, the MFE characteristics of a regime depend sensitively on the quality of the competition between the E- and I-populations. This competition depends in part on the system’s parameters, such as the synaptic coupling strengths and the efficacy of the slow- versus fast- synaptic conductances. In our model we have found that altering these parameters can have strong ramifications for the dynamical properties of the regime. We predict that, in real V1, the pharmacological manipulation of similar quantities should affect the characteristics of MFEs and, in turn, the computational properties of the real cortex (see also Shew et al. 2011).

### Implications for other parts of the nervous system

While our present model is developed specifically to address phenomena observed in V1, some of its dynamical features are likely shared by certain other parts of the brain (and not by others). For example, MFE-like structures may well exist in the dynamics of the leech ganglia, mammalian motor cortex, rat auditory cortex and mammalian hippocampus (Mazzoni et al. 2007; Hatsopoulos et al. 1998; Hahn et al. 2010; Dehghani et al. 2012; Sakata and Harris 2009; Leinekugel et al. 2002; Csicsvari et al. 2000), but probably not in e.g. parts of the hypothalamus and brainstem (Karlsson et al. 2004, 2005; Blumberg 2005). A natural conjecture would be that neuronal networks characterized by flexibility, sensitivity and high gain are more likely to exhibit MFEs.

### Connections to neuronal avalanches

The term ‘avalanche’ generally refers to spiking behavior that involves bursts with pauses in between. The nature of the bursts observed in experiments vary widely, ranging from spontaneous activity involving relatively large numbers of neurons and occurring 1–2 s apart on average (as seen in culture Beggs and Plenz 2003) to smaller and more frequent bursts observed *in vivo* (as in Hahn et al. 2010); the MFEs seen in our ‘biologically plausible’ network regimes resemble more the latter. Power-law distributions of burst sizes and inter-event times are important characteristics of neuronal avalanches; they are in fact part of the definition.

Here, as in (Rangan and Young 2012), we have reserved the term ‘multiple-firing-events’ (MFEs) to refer specifically to sequences of firing-events which are causally linked by recurrent synaptic connections and triggered essentially by single excitatory spikes. Because of the infinitely-small synaptic timescales in our model, MFEs can easily be pinpointed. We have also demonstrated in the Supplementary Material that even as we permit nonzero rise times for fast synaptic currents, these events continue to be identifiable, as they tend to occur within very short time-windows.

As to how exactly neuronal avalanches and MFEs are related: we believe that, though their emphases differ, the phenomena captured by these two approaches—one experimental and the other theoretical—likely have much in common. MFEs require direct causality; avalanches do not, yet some fraction of the experimentally observed firing in avalanches are likely causally linked, given the density and small spatial extent of the electrode arrays used in the recordings. Power-law behavior is a pre-condition for neuronal avalanches; for MFEs it is not, but interestingly, not only do MFEs occur in our ‘biologically plausible’ regimes, their magnitudes tend to have power law distributions. We think this is far beyond coincidence, and that further investigation is needed to both (i) clarify the role played by recurrent synaptic interactions in the avalanches observed experimentally and (ii) understand, on a theoretical level, why power-law distributions of MFE magnitudes in network models tend to produce more realistic outputs.

### Implications for theoretical neuroscience

Since MFEs are not easily captured by rate models or by population dynamics approaches, our results suggest that these analytical tools in their present form may not be adequate. They point to the need for higher order statistics to complement the use of firing rates, including new ways to capture effectively collaborative activity among possibly random groups of neurons. Finally, we hope we have shown that, at least for the regime discussed here, statistics alone do not tell the whole story; behind all that are dynamical events that are causally linked.

## Methods

### Model details

Each of the neurons in our network is modeled using conductance based integrate and fire equations:

$$\begin{array}{@{}rcl@{}} \tau _{V}\frac{d}{dt}V &=& -\left( V-V^{L}\right) -g_{\text{fast}}^{\text{input}} \left( V-V^{E}\right)\\ &&-g_{\text{slow}}^{\text{syn,}E}\left( V-V^{E}\right) -g_{\text{fast}}^{\text{syn,}E} \left( V-V^{E}\right)\\ &&-g_{\text{fast}}^{\text{syn,}I} \left( V-V^{I}\right) , \end{array} $$

with *V* ^*L*^ = 0, *V* ^*E*^ = 14/3, *V* ^*I*^ = −2/3, *τ*_*V*_ = 20 ms (Dayan and Abbott 2001). The voltage *V* of neuron *n* evolves continuously until *V(t)* crosses a membrane-potential threshold *V* ^*T*^ = 1, at which point this neuron fires, and its voltage *V(t)* is reset to *V* ^*T*^ = 0, and held there for a time *τ*_ref_ = 1. The term $g_{\text {slow}}^{\text {syn,}E}$ models the slow excitatory synaptic conductance, and the term $g_{\text {fast}}^{\text {syn,}Q}$ models the fast *Q*-type synaptic conductance, $Q\in \{E,I\}$. The slow-excitatory conductance $g_{\text {slow}}^{\text {syn,}E}$ for the neuron *n* is given by $g_{\text {slow}}^{\text {syn,}E}\left ( t\right ) =\sum _{n^{\prime }}W_{\text {slow}}^{nn^{\prime }}\alpha _{\text {slow}}^{E}\star m_{n^{\prime }}$, where *n′* runs over all E-neurons, $W_{\text {slow}}^{nn^{\prime }}$ describes the coupling strength between neuron *n′* and neuron *n*, *m*_*n′*_ is a sum of delta-functions representing the firing activity of neuron *n′*, and $\alpha _{\text {slow}}^{E}\left ( t\right ) $ is an alpha-function with instantaneous rise-time and a decay-time of $\tau _{\text {slow}}^{E} $ ∼ 50–150 ms. The fast *Q*-type conductances $g_{\text {fast}}^{\text {syn,}Q},\ Q\in \{E,I\}$, are given by similar equations parametrized by $W_{\text {fast}}^{nn^{\prime }}$ and $\alpha _{\text {fast}}^{Q}$, where this latter function has infinitely fast decay $\tau _{\text {fast}}^{Q}\rightarrow 0$. To ensure that this system remains well posed, we take the formal limit as *τ*_*Q*_ and *τ*_ref_ tend to 0^+^. The order in which we take these limits affects the dynamics. We assume that $\tau _{\text {ref}}\gg \tau _{\text {fast}}^{Q}$, so each neuron can only fire once at each instant in time, and $\tau _{\text {fast}}^{I}\sim \tau _{\text {fast}}^{E}$ as $\tau _{\text {fast}}^{E},\tau _{\text {fast}}^{I}\rightarrow 0^{+}$. This assumption allows for a biologically realistic competition between excitatory and inhibitory populations.

The coupling strengths $W_{\text {fast}}^{nn^{\prime }}$ and $W_{\text {slow}}^{nn^{\prime }}$ encode the connectivity of the network, *n′* being the transmitting and *n* the receiving neuron. We assume that the neurons are sparsely connected, so many of these weights will be 0. Since our model incorporates a slow excitatory synaptic current but not a slow inhibitory synaptic current, $W_{\text {slow}}^{nn^{\prime }}=0$ when *n′* is inhibitory. Also, since only excitatory cells broadcast long-range connections, $W_{\text {fast}}^{nn^{\prime }}=W_{\text {slow}}^{nn^{\prime }}=0$ when *n′* is inhibitory and in a different hypercolumn than *n*.

If two neurons are connected, then we assume that their connection strengths depend only on (i) their types, (ii) whether or not they share the same orientation preference, and (iii) whether or not they lie in the same hypercolumn. That is to say, given two connected neurons *n* and *n′* of types *Q* and *Q′* in clusters *C*_*jk*_ and *C*_*j′k′*_ respectively, $W_{\text {fast}}^{nn^{\prime }}$ and $W_{\text {slow}}^{nn^{\prime }}$ depend only on *Q* and *Q′*, *δ*_*jj′*_ and *δ*_*kk′*_. Thus for pairs of neurons with *δ*_*kk′*_ = 1, there are 6 local-coupling parameters, which we denote by $S_{\text {fast}}^{Q,Q^{\prime }}$ and $S_{\text {slow}}^{Q,E}$. If *δ*_*jj′*_ = 1, there are 4 long-range-coupling parameters, which we denote by $L_{\text {fast}}^{Q,E}$ and $L_{\text {slow}}^{Q,E}$. For ease of notation we use *S* ^*QQ′*^ to refer to $S_{\text {fast}}^{Q,Q^{\prime }}$, and *L* ^*Q*^ to refer to $L_{\text {slow}}^{Q,E}$. We will also refer to $\tau _{\text {slow}}^{E}$ as *τ*_slow_.

The term $g_{\text {fast}}^{\text {input}}$ models the excitatory conductance associated with feedforward input to neuron *n*. We consider two general classes of input $g_{\text {fast}}^{\text {input}}$ for this system: (i) an input which models the unstimulated or ‘background’ state of the cortex, and (ii) an input which models a ‘stimulus’ intended to represent a drifting grating of some diameter. Both the background drive and the stimulus to neuron-*n* are modeled by an input of the form $g_{\text {fast}}^{\text {input}}=S_{\text {drive}}^{Q}\sum _{h}\alpha _{\text {fast}}^{E}\left ( t-T_{h}^{\text {drive}}\right )$, where the spike-times $T_{h}^{\text {drive}}$ are chosen from a Poisson process with rate *η*_*Q*, *j*, *k*, *l*_ *(t)* that depends on *Q*, *j* and *k*, as well as on the neuron’s index *l*. In background the rate of this Poisson input is chosen to be constant across all orientations *j* and hypercolumns *k*, as well as all indices *l*. The stimulus is modeled by increasing this Poisson input drive to selected clusters.

For example, in order to model a drifting grating stimulus of orientation *θ*_*j*_ with a moderate spatial diameter, we increase the drive to several clusters *C*_*j1*_, *C*_*j2*_, …. Thus, in general, the drive is given by a cluster-independent background drive plus a cluster-dependent stimulus- specific drive:

$$ \eta _{Q,j,k,l}\left( t\right) =\eta _{Q}^{\text{bkg}}+\eta _{Q,j,k,l}^{\text{stim}}\left( t\right) . $$

We simulate the type of input a ‘simple’-like cell would receive under a drifting-grating stimulus by setting

$$ \eta _{Q,j,k,l}^{\text{stim}}\left( t\right) =C_{Q,j,k}\eta _{Q}^{\text{bkg}}\left( 1+\sin \left( \omega \left( t-\phi _{l}\right) \right) \right) . $$

In this expression *C*_*Q*, *j*, *k*_ represents the effective contrast/strength of the stimulus as perceived by the neurons in question, and is high only for those clusters *j*, *k* which would be influenced given the orientation and size of the drifting-grating; $C_{E,k,j}=3C_{I,k,j} $ to simulate the excess of orientation-tuned input to the excitatory population which may arise from feedback. The frequency *ω* = 4 Hz is the temporal-frequency of the grating, and the phase *ϕ*_*l*_ is related to the spatial-phase preference of the cell, and is randomly distributed uniformly across the neurons within each cluster. We note that our main conclusions do not change when we ignore the spatial phase-preference and take *η* ^stim^ *(t)* to be a constant.

For our simulations we use a network with 8 hypercolumns each with 3 orientation domains, resulting in a total of 24 ‘clusters’ each containing a few hundred excitatory and inhibitory neurons. Connectivity within each cluster is random and ∼20 % for E- and ∼ 50 % for I-neurons, as I-neurons are supposed to arborize more densely. Connectivity across clusters are about half that within the cluster. A typical value of $\tau _{\text {slow}}^{E}$ used is 128 ms.

Further details on model description are given in Supplementary Material.

### Benchmarking

Our model as described above has ∼ 10 free parameters corresponding to short- and long-range synaptic coupling strengths, *S* ^*QQ′*^, *L* ^*QQ′*^ respectively, and background drive. Such a high-dimensional space cannot yet be systematically searched, and the nonlinear dynamic phenomena we were looking for could not easily be captured by any sort of ‘gradient descent’ in parameter space. As a first step in our constraining procedure, we restricted ourselves to parameter values that produced results consistent with known physiological data such as EPSPs and background E- and I-firing rates in V1. We then pitched competing sets of parameters against one another (e.g., *S* ^*EE*^ versus *S* ^*EI*^ + *S* ^*IE*^), and performed some guided parameter sweeps, i.e., at each step, we performed a preliminary dynamical analysis of the search results, and used that to guide the direction of the search, moving closer successively to parameter regions that exhibited the required characteristics.

We mention that among the more delicate parameters are those related to the Poisson background drive, i.e., $S^Q_{\mathrm {drive}}$ and $\eta ^{\mathrm {bkg}}_Q, Q = E, I$. These parameters (along with the coupling strengths) determine not only the firing rates but also the quality of the competition between the E- and I-populations. For example, if $S^E_{\mathrm {drive}} \cdot \eta ^{\mathrm {bkg}}_E$ is too low, then our system will not fire sufficiently frequently in background. To put the regime in a position of high gain, it is necessary that $S^E_{\mathrm {drive}}$ be not too large and $\eta ^{\mathrm {bkg}}_E$ be not too small; in our case $S^E_{\mathrm {drive}}$ is approximately 0.5*S* ^*EE*^ and *η* ^bkg^ ∼ 250–500 Hz.

Among the phenomena used for benchmarking, the spontaneous correlated background activity imposed the most serious constraints on our model. In general, features of background patterns depend more sensitively on parameters than features of strongly driven systems, reflecting possibly the corresponding underlying sensitivity in the real V1 (see, e.g., Ecker et al. 2010).

Detailed discussions of the restrictions imposed by each of phenomena (1)–(4) are given in Supplementary Material.

### Calculating cross-covariance

As a diagnostic for spontaneous background patterns, we measured the cross-covariance of firing and subthreshold-voltage activity between cells in the same and/or different orientation domains and hypercolumns, and we define these measurements here. Let *m*_*Q*, *j*, *k*, *l*_ denote the times at which the *l*th neuron of type *Q* in cluster *C*_*jk*_ fires. Cross-covariances can be summarized in the functions

$$\begin{array}{lll} &&C\left( Q,Q^{\prime },A,B,\tau \right)\\ &&\quad =dt\sum\limits_{j,k,l,j^{\prime},k^{\prime },l^{\prime}}m_{Q,j,k,l}\left( t\right) m_{Q^{\prime },j^{\prime },k^{\prime},l^{\prime }}\left( t-\tau \right) \ . \end{array} $$

Here $Q,Q^{\prime }\in \left \{ E,I\right \} $, and the sum over *j′* is taken over $j^{\prime }|\delta _{jj^{\prime }}=A$, and the sum over *k′* is taken over $k^{\prime }|\delta _{kk^{\prime }}=B$. The variable *A* in this definition indicates whether the crosscovariance refers to either neurons with the same orientation (*A* = 1) or different orientations (*A* = 0), and the variable *B* indicates whether the crosscovariance sums over neurons within the same hypercolumn (*B* = 1) or different hypercolumns (*B* = 0). Similarly, we can compute the ‘spike-triggered-average’ of the voltage distribution:

$$\begin{array}{lll} &&STV\left( Q,Q^{\prime },A,B,\tau ,V\right)\\ &&\quad=dt\sum\limits_{j,k,l,j^{\prime },k^{\prime },l^{\prime}}m_{Q,j,k,l}\left( t\right) \delta\left( V {} - {} V_{Q^{\prime },j^{\prime},k^{\prime },l^{\prime }}\left( t-\tau \right) \right) \text{,} \end{array} $$

Where, again, the sums over *j′* and *k′* are determined by *A* and *B*. In order for our model to exhibit a background regime which is physiologically reasonable, we would expect $STV\left( E,Q^{\prime},1,0,\tau ,V\right)$ to be elevated (i.e., have a mean closer to *V*_*T*_) with respect to $STV\left( E,Q^{\prime },0,0,\tau ,V\right) $ for *Q′* = *E*, *I*. Moreover, we would expect $C\left( E,Q^{\prime },1,0,\tau \right)$ to be elevated with respect to $C\left( E,Q^{\prime },0,0,\tau \right)$ for $Q^{\prime }\in \left \{ E,I\right \} $. This elevation should last from ∼ 50 to ∼ 500 ms, defining an emergent timescale $\tau _{\text {persist}}^{\text {bkgrnd}}$. Plots of cross-covariances are shown in Fig. 2. Note that $\tau _{\text {persist}}^{\text {bkgrnd}}\sim $ 300 ms.

### Binning of MFEs

While in principle MFEs occur mostly instantaneously when infinitesimal synaptic timescales are used, we have found it convenient in practice to summarize spiking activity using 1 ms timebins, and to call *k* spikes in a single bin an MFE of magnitude *k* provided *k* spikes/ms is significantly higher than mean firing rate for the population. For a local population with relatively low firing rates (as in our model), we have found this to be a reasonable operational definition, in the sense that MFE characteristics are not substantially affected by bin sizes as they range from a fraction of a ms to a few ms.

## Electronic supplementary material

Below is the link to the electronic supplementary material.

(PDF 953 kb)
