# Supplementary material for: Emergent dynamics in a model of visual cortex
Source: J Comput Neurosci. 2013 Mar 22;35(2):155–67. doi: 10.1007/s10827-013-0445-9 (PMC3766520; doi:10.1007/s10827-013-0445-9)
Supplement: Supplementary file 1 — (PDF 953 kb) [file 10827_2013_445_MOESM1_ESM.pdf]

Emergent dynamics in a model of visual cortex: Supplementary Material

Aaditya V. Rangan, Lai-Sang Young

December 8, 2012

1 Model Description

The model discussed in this section is that of a 2mm^2 patch of the visual cortex. To link macroscopic phenomena to neuron-to-neuron interactions, we have built our model out of a network of spiking point neurons, using the voltages and conductances of individual neurons as microscopic variables.

Our network's architecture reflects some basic physiological features of V1, such as orientation domains and receptive fields, and is constrained by neurophysiological data (e.g., connection sparsity and synaptic strengths [1–10]). Even though the network is large and contains many thousands of neurons, it is not overly detailed, and we have included only a dozen or so of the most important parameters, such as short- and long-range coupling strengths between neuronal populations, and strengths of feedforward input currents. Additionally, we have chosen several well-studied V1 phenomena which impose further constraints on our model parameters [11–34]. These phenomena include, but are not limited to, (i) the structured spatial and temporal correlations observed in the absence of stimulus [35–40]; (ii) the steady state firing-rate responses associated with high-contrast drifting-grating stimuli and (iii) the suppression of these responses seen when the spatial extent of these gratings is increased [41–43].

Taken together, the collection of phenomena above constitutes a substantial constraint on the dynamics of our model. Given the relatively small number of free parameters, it was by no means guaranteed that our model would be able to satisfy all of these benchmarks for *any* choice of parameters. Our principal result, as demonstrated below, is that there are fairly sizable regions in parameter space for which our model exhibits simultaneously all of the phenomena studied, even as some of these phenomena appear to place conflicting demands on the system.

As explained above, we would like to work toward a model that can provide a window into real V1 dynamics. At the same time, we designed our model with an eye towards clarifying the underlying dynamical processes. Such a model must necessarily involve a fair amount of idealization, both in network architecture and in its microdynamics; this will be evident in the model description to follow. Our challenge was to idealize enough to obtain a model that can potentially be dissected and analyzed, but not too much so as to interfere with biologically relevant mechanisms.

The main features of our model are introduced in Sects. 1.1 and 1.2. The first restrictions on the 20 or so parameters that appear in this model are discussed in Sect. 1.3.

1.1 Network architecture, equations and parameters

Our model is intended to represent a small patch of layer 2/3 of V1, comprising a handful of hypercolumns each with multiple orientation domains; within the cat such a patch would have a diameter of $\sim$ 2mm. In order to emphasize the phenomena of orientation selectivity and patterned background activity, we structure our model so that each orientation domain in a hypercolumn is represented by a single 'cluster' containing both excitatory and inhibitory cells. There are J orientation domains within each hypercolumn, corresponding to J different orientations ($\theta_j = j\pi/J$), and there are K hypercolumns in total. Thus, the cluster C_{jk} corresponds to the jth orientation domain of hypercolumn k.

The excitatory and inhibitory cells within each cluster are connected to one another sparsely and randomly, both within and across the orientation domains of a given hypercolumn. The excitatory cells are also sparsely

connected to orientation domains of similar orientation-preference within other hypercolumns. We will refer to connections between cluster C_{jk} and clusters $C_{j'k}$ (i.e., clusters within the same hypercolumn) as *local* connections, and connections between C_{jk} and $C_{jk'}, k' \neq k$, as *long-range* connections. See Fig 1 in the main text for an illustration of our network architecture.

Each cluster has N_E excitatory and N_I inhibitory neurons. In order to focus clearly on causal neuron-neuron interactions we model each neuron using single-compartment leaky integrate-and-fire equations in which the state-variables are membrane-potential V and a slowly decaying excitatory synaptic conductance $g_{\text{slow}}^{\text{syn},E}$ (intended to represent NMDA-type excitation [61–70]). We also include typical fast synaptic currents associated with AMPA-type excitation and GABA–A type inhibition, but these currents are modeled with instantaneous rise and infinitely fast decay, thus obviating the need for additional state-variables to track these quantities. We have made this simplification within our model in order to clearly distinguish the fast-synaptic dynamics from the slower dynamics associated with leakage currents and slow-synaptic currents, thus accentuating the causal relationship between a cell and its post-synaptic targets. Nevertheless, our major results remain valid even when the fast-synaptic time-scales are not infinitely short (see, e.g., section 2.1).

The state-variable V of a typical neuron n evolves according to a set of ODEs which can be described as follows:

$$\tau_V \frac{d}{dt} V(t) = -\left(V - V^L\right) - g_{\text{fast}}^{\text{input}}(t)\left(V - V^E\right) \quad (1)$$

$$-g_{\text{slow}}^{\text{syn},E}(t)\left(V - V^E\right) - g_{\text{fast}}^{\text{syn},E}(t)\left(V - V^E\right) - g_{\text{fast}}^{\text{syn},I}(t)\left(V - V^I\right). \quad (2)$$

In these equations $V^L = 0$, $V^E = 14/3$, $V^I = -2/3$ (expressed in non-dimensional units) are reversal potentials associated with the leakage-, excitatory- and inhibitory-currents, and the leakage-timescale $\tau_V =$ 20ms [71]. The voltage V of neuron n evolves continuously until $V(t)$ crosses a membrane-potential threshold $V^T = 1$, at which point this neuron fires, and its voltage $V(t)$ is reset to $V^L = 0$, and held there for a time τ_{ref}.

The term $g_{\text{fast}}^{\text{input}}$ models the excitatory conductance associated with feedforward input to neuron n (see Eq 5 below). The term $g_{\text{slow}}^{\text{syn},E}$ models the slow excitatory synaptic conductance, and the term $g_{\text{fast}}^{\text{syn},Q}$ models the fast Q-type synaptic conductance, $Q \in \{E, I\}$.

The slow-excitatory conductance $g_{\text{slow}}^{\text{syn},E}$ for the neuron n is given by

$$g_{\text{slow}}^{\text{syn},E}(t) = \sum_{n' \text{ of type } E} W_{\text{slow}}^{nn'} \alpha_{\text{slow}}^{E} \star m_{n'}, \quad (3)$$

where $W_{\text{slow}}^{nn'}$ describes the coupling strength between neuron n' and neuron n, $m_{n'}$ is a sum of delta-functions representing the firing activity of neuron n', and $\alpha_{\text{slow}}^{E}(t)$ is an alpha-function with instantaneous rise-time and a decay-time of $\tau_{\text{slow}}^{E} \sim 50 - 150$ms.

In a similar fashion, the fast Q-type conductance $g_{\text{fast}}^{\text{syn},Q}$, $Q \in \{E, I\}$, is given by

$$g_{\text{fast}}^{\text{syn},Q} = \sum_{n' \text{ of type } Q} W_{\text{fast}}^{nn'} \alpha_{\text{fast}}^{Q} \star m_{n'}. \quad (4)$$

The alpha-function $\alpha_{\text{fast}}^{Q}(t)$ has instantaneous rise-time and an infinitely fast decay-time of $\tau_{\text{fast}}^{Q} = 0$. Thus, each time a neuron fires, the fast synaptic-conductances create an instantaneous jump in the voltage of the postsynaptic neuron and multiple neurons can be driven to fire at any instantaneous point in time.

Now we must emphasize that, due to the singular nature of Eq 4 (as well as Eq 5 below), the Eq 1 is not actually a well-posed ordinary differential equation. We model the instantaneous currents $g_{\text{fast}}^{\text{syn},Q}$ by starting with fast time-scales τ_{fast}^{Q} which are not zero, and then taking the formal limit as these time-scales, as well as τ_{ref}, tend to 0^+. The order in which we take these limits affects the dynamics. We assume that $\tau_{\text{ref}} \gg \tau_{\text{fast}}^{Q}$, so each neuron can only fire once at each instant in time, and $\tau_{\text{fast}}^{I} \sim \tau_{\text{fast}}^{E}$ as $\tau_{\text{fast}}^{E}, \tau_{\text{fast}}^{I} \to 0^+$. This assumption allows for a biologically realistic competition between excitatory and inhibitory populations.

The coupling strengths $W_{\text{fast}}^{nn'}$ and $W_{\text{slow}}^{nn'}$ encode the connectivity of the network, n' being the transmitting and n the receiving neuron. We assume that the neurons are sparsely connected, so many of these weights

will be 0. Since our model incorporates a slow excitatory synaptic current but not a slow inhibitory synaptic current, $W_{\text{slow}}^{nn'} = 0$ when n' is inhibitory. Also, since only excitatory cells broadcast long-range connections, $W_{\text{fast}}^{nn'} = W_{\text{slow}}^{nn'} = 0$ when n' is inhibitory and in a different hypercolumn than n.

If two neurons are connected, then we assume that their connection strengths depend only on (i) their types, (ii) whether or not they share the same orientation preference, and (iii) whether or not they lie in the same hypercolumn. That is to say, given two connected neurons n and n' of types Q and Q' in clusters C_{jk} and $C_{j'k'}$ respectively, $W_{\text{fast}}^{nn'}$ and $W_{\text{slow}}^{nn'}$ depend only on Q and Q', $\delta_{jj'}$ and $\delta_{kk'}$. Thus for pairs of neurons with $\delta_{kk'} = 1$, there are 6 local-coupling parameters, which we denote by $S_{\text{fast}}^{Q,Q'}$ and $S_{\text{slow}}^{Q,E}$. If $\delta_{jj'} = 1$, there are 4 long-range-coupling parameters, which we denote by $L_{\text{fast}}^{Q,E}$ and $L_{\text{slow}}^{Q,E}$. For ease of notation we will later use $S^{QQ'}$ to refer to $S_{\text{fast}}^{Q,Q'}$, and L^Q to refer to $L_{\text{slow}}^{Q,E}$. We will also refer to $g_{\text{slow}}^{\text{syn},E}$ as g_{slow} and τ_{slow}^{E} as τ_{slow}.

1.2 Two types of input

We will consider two general classes of input $g_{\text{fast}}^{\text{input}}$ for this system: (i) an input which models the unstimulated or 'background' state of the cortex, and (ii) an input which models a 'stimulus' intended to represent a drifting grating of some diameter. Both the background drive and the stimulus to neuron-n are modeled by an input of the form

$$g_{\text{fast}}^{\text{input}} = S_{\text{drive}}^{Q} \sum_h \alpha_{\text{fast}}^{E} \left(t - T_h^{\text{drive}}\right) \ , \tag{5}$$

where the spike-times T_h^{drive} are chosen from a Poisson process with rate $\eta_{Q,j,k,l}(t)$ that depends on Q, j and k, as well as on the neuron's index l. In background the rate of this Poisson input is chosen to be constant across all orientations j and hypercolumns k, as well as all indices l. The stimulus is modeled by increasing this Poisson input drive to selected clusters. For example, in order to model a drifting grating stimulus of orientation θ_j with a small spatial diameter, we increase the drive to the jth cluster in a single hypercolumn [1]. If, on the other hand, we wish to model a drifting grating stimulus of orientation θ_j with a very large spatial diameter, we would increase the drive to the jth cluster of every hypercolumn. Thus, in general, the drive, i.e., the rate of the Poisson input, to each cluster is given by a cluster-independent background drive plus a cluster-dependent stimulus-specific drive:

$$\eta_{Q,j,k,l}(t) = \eta_Q^{\text{bkg}} + \eta_{Q,j,k,l}^{\text{stim}}(t) \, .$$

We simulate the type of input a 'simple'-like cell would receive under a drifting-grating stimulus by setting

$$\eta_{Q,j,k,l}^{\text{stim}}(t) = C_{Q,j,k}\eta_Q^{\text{bkg}} \left(1 + \sin\left(\omega\left(t - \phi_l\right)\right)\right) .$$

In this expression $C_{Q,j,k}$ represents the effective contrast of the stimulus as perceived by the neurons in question, and is high only for those clusters j, k which would be influenced given the orientation and size of the drifting-grating. We choose $C_{E,k,j} = 3C_{I,k,j}$ to simulate the excess of orientation-tuned feedforward input to the excitatory population which may arise from feedback from other layers [33]. The frequency ω (which we take to be 4Hz) is the temporal-frequency at which the grating passes over the cell's receptive field center, and is related to the spatial-frequency of the grating, as well as the grating's drift speed. The phase ϕ_l is related to the spatial-phase preference of the cell, and is randomly distributed within each cluster [8, 69, 73, 74]. We will not consider the distinction between simple and complex cells, except to note that our main conclusions do not change when we take $\eta^{\text{stim}}(t) = C$ [75].

1.3 Constraints from physiology

The following are parameters in our model:

(a) network size (J, K, N_Q),
(b) sparsity of connections,

[1] Within the real V1, cells in any given orientation-hypercolumn tend to have overlapping receptive fields of $\sim 1^\circ$, and localized visual stimulus typically directly stimulates $\sim 1-2$ adjacent hypercolumns [7, 72]. Our model is designed to reflect this physiology; we model a localized drifting grating stimulus by increasing the input drive to all the neurons in $1-2$ hypercolumns with the appropriate orientation preference.

(c) synaptic coupling strengths ($S^{QQ'}_{\text{fast}}$, $S^{QQ'}_{\text{slow}}$, $L^{Q,E}_{\text{fast}}$, $L^{Q,E}_{\text{slow}}$),
(d) time-scale of slow-excitation τ_{slow}, and the ratio $\tau^{I}_{\text{fast}}/\tau^{E}_{\text{fast}}$,
(e) input (S^{Q}_{drive}, η^{bkg}_{Q}, $\eta^{\text{stim}}_{Q,j,k}$).

Many of these parameters are constrained in one way or another by the physiology and architecture of the real V1 as we now discuss.

(a) and (b): With regard to network size and connectivity, a typical small patch of layer 2/3 of V1 with a radius comparable to an ocular dominance column comprises $\sim 5-10$ hypercolumns, each comprising a continuum of orientation domains [7, 22, 76, 77]. To grossly capture the facts that (i) each orientation domain is influenced locally by domains of differing orientation preference, and (ii) each orientation domain is influenced laterally by multiple nearby orientation domains of similar orientation preference, we choose $K = 8$ and $J = 3$. Our results do not change qualitatively if we include more hypercolumns or resolve orientation-space more finely [2]. One feature not incorporated is the spatial structure of the long-range connections across scales of several mm — in our model long-range connections between pairs of hypercolumns are statistically identical. A typical orientation-domain within layer 2/3 of cat V1 has ~ 700 excitatory cells and ~ 200 inhibitory cells [5, 78–81] [3] and many types of inhibitory neurons are known to arborize densely [82–85]. We use $N_E = N_I = 128$ for the simulations and parameter-sweeps presented in this section; too small an N_Q (e.g. $N_Q = 8$) will not produce a dynamic regime with statistics that are physiologically acceptable. We take N_I comparable to N_E to reflect the fact that inhibitory neurons arborize more densely The regimes (and underlying mechanisms) discussed here persist for larger values of N_Q. For an example, see section 4.3. Experiments also indicate that excitatory neurons are not connected together in an all-to-all fashion [32]. We use a network connectivity of about 25%.

(c) and (d): The synaptic strengths are constrained by several electrophysiological experiments within V1, which indicate that typical EPSPs and IPSPs are not much larger than 1.5mV [4, 9, 86, 87]. Thus a typical cell near reset will require $\sim 10-20$ synaptic excitatory 'kicks' (in quick succession) to be driven over threshold. This constraint places an upper bound on the local and long-range coupling strengths $S^{QQ'}_{\text{fast}}$ and $L^{Q,E}_{\text{fast}}$. The time-integrated slow-excitatory EPSPs observed in the cortex are not much larger than the time-integrated fast EPSPs [64, 68, 70, 88]; that too will impose constraints in our parameter choices. The time-scale of slow-excitation in V1 varies from $\sim 40-120$ms [61, 63, 65]. In our model we use $\tau^{E}_{\text{slow}} = 128$ms for most of our simulations, but this choice is not critical to our results. With regard to the fast time scales, the ratio $\tau^{I}_{fast}/\tau^{E}_{fast}$, which determines the order in which events are resolved, is taken to be 1; our results are not especially sensitive to this ratio provided it is not too large or too small.

(e): The input to our system is similarly constrained by experiments. Within the real V1 the EPSPs due to input are rarely large, and consist of many micro-EPSPs as well as many feedback and feedforward connections from other layers [89]. Moreover, it is observed that when V1 is driven by stimulus, a large portion of the feedforward input is orientation-tuned and may also preferentially target the excitatory population [33, 90]. Thus, we constrain our system by requiring that (i) the magnitude of S^{Q}_{drive} is small (say, $< S^{Q,E}_{\text{fast}}$), and (ii) the additional input associated with an oriented visual stimulus should affect the excitatory population more than the inhibitory population (i.e., $\eta^{\text{stim}}_{E,j,k} > \eta^{\text{stim}}_{I,j,k}$ via $C_{E,j,k} > C_{I,j,k}$ when cluster j,k is stimulated).

In V1 it is observed that typical dynamic regimes in background produce excitatory firing-rates of a few Hz, with inhibitory firing-rates that are not much higher [27, 91–93]. This places a lower bound on the input

[2] While our discretization into $J = 3$ orientation domains per hypercolumn is somewhat of an idealization, the local interaction between these clusters qualitatively captures the continuum of orientation-domains in V1, and we are not sure if a choice of $J > 3$ would be substantially more realistic. Specifically, while the division of V1 into orientation domains is well documented, the local architecture associated with these domains is not as well documented. The 'messiness' in the local architecture seems far greater than that assumed in a linearly parametrized ring model [32]. Moreover, the orientation tuning seen in many cells in V1 is quite broad [96, 134], with a width at half-height comparable to pi/3. Thus, there are probably no more than $4-7$ clearly distinguishable orientation domains in the real V1. Our choice $J = 3$ seems like it can coarsely capture the 'activated', and 'inactivated' sections of any given hypercolumn under strong grating stimulus, while allowing us to coarsely canvass the emergence of background patterns. Increasing J in our model would require the introduction of extra parameters, which we did not feel could be appropriately constrained.

[3] There are roughly 40,000 neurons per mm^3 in the cat V1, and layer 2/3 is about 0.38mm thick. Thus, there are around 14,000 cells per mm^2 in layer 2/3. There are between 4-7 orientation pinwheels per mm^2 in the cat, so there are around 2800 neurons per orientation pinwheel. With each pinhweel divided into 3 orientation domains, there would be roughly 700 excitatory neurons and 200 inhibitory neurons per orientation domain.

current to our system. If the combination $S^E_{\text{drive}} \cdot \eta^{bkg}_E$ is too low, then our system will not fire sufficiently frequently in background. Also, even though our model is structured specifically to allow many neurons to fire concurrently, we do not consider parameter regimes which admit synchronous firing or firing events that involve too a large fraction of the population, as that is unbiological. See Fig 1.

Last but not least, many different experiments have demonstrated that V1 typically operates in a regime of high-gain [44–60]. We build that into our model by requiring that the background input to the system places the network in a state that can be easily excited by stimulus. Note that, unlike the restriction associated with background firing-rate (which constrains $S^E_{\text{drive}} \cdot \eta^{bkg}_E$), the restriction associated with a high-gain regime places an upper bound on S^E_{drive}. If S^E_{drive} is too large, then the system will respond linearly as $\eta_{E,j,k}$ increases [94]. Within a high gain regime S^E_{drive} is small, $\eta_{E,j,k}$ is relatively large, and each neuron receives many relatively weak input EPSPs. This type of input ensures that the membrane-potential of each neuron is often close to V^T, and that a small additional input can drive many neurons to fire.

How good is the model described above? One way to find out is to try to replicate some of the empirical observations of V1 that are not direct consequences of the modeling, i.e., they are not directly implied by our model architecture or by the rules of dynamics. The following sections discuss several such empirical observations. In each case, we will demonstrate (via simulations) that the phenomenon occurs for suitable choices of parameters, give a sense of how they constrain the network, and discuss briefly the main features of the dynamical regimes. Our aim is to produce, at the end of section 5, regions in parameter space each point of which corresponds to a model which exhibits all of the phenomena considered.

Recall that $S^{QQ'}_{\text{fast}}$ is abbreviated as $S^{QQ'}$, and L^{QE}_{slow} as L^Q.

2 Dynamics: generation of MFEs

2.1 Fast time scales and MFEs

In the main text of this paper we often refer to MFEs (multiple firing events), which are idealizations of a concept that can only be defined precisely in the limit $\tau_E \to 0$, for in this limit, no other forces can intervene between the start and completion of a synaptic event. Taking τ_E to be a small but strictly positive number will introduce a small amount of fuzziness, in that it may not always be possible to determine whether a spiking is 'directly caused' by another spike. As long as τ_E and τ_I are significantly smaller than the other timescales in the system, multiple firing events should remain clearly identifiable, if not precisely defined. In Fig 2 we show that MFEs can manifest within more realistic clusters of conductance-based LIF neurons for which the fast synaptic conductances have timescales $\tau_E, \tau_I > 0$.

2.2 Conduction velocity and synaptic delays

The horizontal conduction velocity in mammalian visual cortex has been measured to lie between 0.1 and 0.4 mm/ms, with a mean of roughly 0.3 mm/ms. In other words, the delay due to horizontal transmission is roughly $3 - 10$ ms/mm [4, 18, 97, 98].

For simplicity, the model we discuss in the main text does not include any transmission delay between hypercolumns, nevertheless our main results hold even if we were to include such a delay. Specifically, the dynamic regime of our model continues to generate plenty of MFEs even after we incorporate transmission delays of $5 - 10$ ms between hypercolumns (note that with the relatively small spatial extent of our system different hypercolumns are $\sim 1 - 2$mm apart). The reason why our results are robust with regards to transmission delay is that, in the regime we discuss in the main text, the majority of the MFEs result from fast synaptic interactions within individual orientation domains. The long-range horizontal connections are important for setting the mean level of input to the clusters, but play a subsidiary role in the actual nucleation and execution of most MFEs.

Shown in Fig 3 is a dynamic regime associated with our model when we incorporate long-range transmission delays of 9ms between hypercolumns. The coupling strengths of the system have been adjusted by $\pm 10\%$ so that the regime still retains the same dynamical properties as discussed in the main text. The individual orientation domains still generate MFEs, and the MFEs are still responsible for the large-scale dynamic phenomena (e.g., spontaneous background patterns and surround suppression).

3 Orientation selectivity and localized receptive field

3.1 Two basic features in real V1 and in our model

Orientation selectivity

A large fraction of the excitatory and inhibitory cells in real V1 are orientation-selective, and V1 itself is organized into iso-orientation domains [10, 96, 99–101]. Each iso-orientation domain is ~ 0.25mm in diameter and is modeled in our network via a single cluster C_{jk}. Two commonly observed features of orientation selectivity are that (i) excitatory cells tend to be sharply tuned for orientation, and (ii) inhibitory cells tend to be weakly to moderately tuned for orientation. We will try to capture the gross phenomenon of orientation selectivity in our model in the following way: we assume that a parameter regime exhibits physiologically reasonable orientation selectivity if (i) a stimulation of a subset of clusters corresponding to a given orientation (say, θ_1) does not increase the excitatory firing-rates within the other orientations (i.e., $\theta_2, \ldots, \theta_J$) above their unstimulated background level, and (ii) when a particular orientation is stimulated, the inhibitory neurons associated with that orientation fire more than the inhibitory neurons associated with other unstimulated orientations.

Localized receptive fields

In real V1, it is a fact that presentation of a grating at a cell's preferred orientation, but away from its receptive field center, will not produce additional spiking activity; there may be associated subthreshold activity but it does not give rise to firing-events [33, 104, 105]. A commonly held assumption which can rationalize this phenomenon is that the connectivity within V1 is dominated by local circuitry, implying that activation of the lateral/long-range circuitry alone will not be sufficient to cause firing events (although long-range excitation can give rise to a measurable increase in the average subthreshold voltage of areas far from the stimulated site — see [23, 33, 106]). For our model, this translates into the following: we assume that a parameter regime exhibits localized receptive fields in a physiologically reasonable manner if, when a particular cluster is stimulated, excitatory neurons in other clusters corresponding to the same orientation are not driven to fire appreciably more than they would in background.

3.2 Sample results and constraints on network parameters

To illustrate the ideas above, we first present a sample of simulation results showing the excitatory and inhibitory firing rates in a cluster we call C_{jk}, i.e., a cluster with orientation j and located in hypercolumn k.

	unstim	stim C_{jk}	stim $C_{jk'}$	stim $C_{j'k}$
$[E]_{jk}$	2.7	31	1.7	1.0
$[I]_{jk}$	6.6	36	12	5.4

Caption for Table 1. Here we present steady-state excitatory and inhibitory firing-rates for a typical cluster C_{jk} in our model, recorded under a variety of stimulus paradigms. 'Unstim' means no cluster is stimulated. 'Stim C_{mn}' means C_{mn} is the only cluster stimulated, and in the data shown, $j' \neq j,\ k' \neq k$. In each case involving stimulation the contrast (or stimulus magnitude) is relatively low, and the responses of our system have not saturated.

The data shown illustrate the following: Both excitatory and inhibitory firing in C_{jk} are elevated significantly when C_{jk} is directly stimulated (from 2.7 to 31 for E, from 6.6 to 36 for I). Excitatory firing rate in C_{jk} when $C_{j'k},\ j' \neq j$, is stimulated is significantly lower than when no cluster is stimulated (1.0 vs 2.7), illustrating the fact that excitatory neurons are sharply tuned for orientation. Inhibitory firing rate in C_{jk} when $C_{j'k}$ is stimulated is lower than when $C_{j,k}$ itself is stimulated (5.4 vs 36), illustrating the fact that inhibitory neurons are weakly tuned. Finally, excitatory and inhibitory firing rates in C_{jk} in background are not significantly lower than when $C_{jk'}$, a cluster of the same orientation but in a different hypercolumn, is stimulated (3,7 vs 2,12), illustrating the idea of localized receptive fields [107].

We discuss next how the properties discussed above restrict network parameters:

With respect to connectivity, they tell us that, within a hypercolumn, there must be connections across orientation domains; at the same time, these connections should be no denser than within a cluster. This is the case in our model.

With respect to constraints on synaptic couplings, the properties discussed in this section do not impose strong constraints on our system beyond those already imposed at the end of Section 1, and they are mild compared to those to be imposed in the next two sections. Mainly, the orientation selectivity properties above suggest that local inhibition extends to other clusters within the same hypercolumn, whereas local excitation alone cannot cause other clusters within the same hypercolumn to fire. This suggests that S^{EE} should not be large relative to S^{EI} and S^{IE}. The receptive field property suggests that (i) direct stimulation affects E-neurons more than it does E-neurons in other hypercolumns, i.e. L^E cannot be too large relative to L^I, and (ii) the net effect of long-range excitation should be suppressive, i.e. $L^I > L^E$. These restrictions are not severe; they make the viable parameter corridor in panel [A] of Fig 1 slightly narrower, and impose no additional conditions on the region in panel [B] of Fig 1.

3.3 Sharp tuning

Finally, we comment on an an additional phenomenon regarding orientation tuning − sometimes referred to as ‘sharpening’. The orientation tuning observed in the real V1 is rather sharp and various theoretical investigations have pointed out that this sharp tuning can manifest even when the feedforward inputs into V1 are not sharply tuned. Moreover, modeling work has indicated that the recurrent connectivity within V1 itself may be sufficient to give rise to such a phenomenon (see, e.g., [138]). Our model, as it currently stands, only coarsely resolves orientation space and is not quite detailed enough to clearly investigate this sharpening; we cannot easily disambiguate sharply tuned inputs/responses from broadly tuned inputs/responses.

Here we show that, by increasing the number of orientations in our model, we can indeed qualitatively capture sharpening. We present an example in which we increase J from 3 to 4, and retune the local coupling strengths to maintain the same type of regime discussed in the main text. This $J = 4$ model has fewer inhibitory cells per cluster (64 instead of 128), but otherwise has the same architecture as the $J = 3$ model we discuss in the rest of the paper. In terms of the dynamical regime, we retune the coupling strengths to ensure that (i) MFEs still manifest, and (ii) these MFEs still give rise to spontaneous background patterns, surround suppression, the appropriately structured LFP, etc. As shown in Fig 4A, the MFE distribution for this model has a long tail – MFEs of small to moderate size are typical.

With this new $J = 4$ model we can present weakly tuned input, and measure how sharply tuned the output firing rates are. More precisely, we choose one orientation, say, θ_3, and stimulate 3 clusters of this orientation (i.e., we choose $\eta^{\text{stim}}_{Q,3,k,l}$ as described in section 1.2 with strength $C_{E,3,k} = 0.75$ and $C_{I,3,k} = 0.25$, for $k = 1, 2, 3$, and with strength 0 for $k > 3$). We then stimulate the first 3 clusters of the ‘adjacent’ orientations θ_2 and θ_4 half as strongly (i.e., with $C_{E,2,k} = C_{E,4,k} = 0.375$ and $C_{I,2,k} = C_{I,4,k} = 0.125$ for $k = 1, 2, 3$). We do not stimulate the final ‘orthogonal’ orientation θ_1 at all − the only stimulus received by θ_1 is the background η^{bkg}_Q. This input is broadly tuned, as can be seen in the Fig 4B. We then measure the time-averaged output firing-rates across the first three clusters of each orientation. This data is also shown in Fig 4B, and is much more sharply tuned than the input. This behavior is typical for this model, and persists when we change the number of clusters stimulated or the overall strength of the stimulus.

4 Spontaneous correlated background activity

It has been observed in several mammals (e.g., ferret, cat, and monkey) that, when unstimulated, i.e., in background, the activity in the visual cortex is not homogeneous, but has spatial and temporal structure. The spatial correlations are on the order of 1-2mm and the temporal correlations are on the order of 50-500ms [35–37, 108, 109]. It has also been found that, much of the time, the distinct cortical regions which exhibit correlated behavior are orientation domains of similar orientation preference separated by $\sim 0.5 - 2$mm [23, 35].

4.1 Model regimes exhibiting realistic V1 background activity

By interpreting a collection of experiments [33, 35–40, 108], we propose that the following dynamic picture of our network correctly represents the phenomenon observed: When unstimulated, our network should admit epochs of sustained activity across a collection of orientation domains with equivalent orientation, i.e., a collection of clusters $\{C_{jk}, j = \theta\}$ for some θ. When a pattern is activated, the sustained activity corresponds to increases in both mean firing-rate and mean subthreshold voltage, and clusters with a different θ are usually not concurrently activated during this period (though some concurrent patterns are permitted from time to time). Any given sustained activity pattern can persist for $\sim 50 - 500$ms before either decaying to a homogeneous state or evolving into another sustained pattern associated with a different θ. Fig 2 in the main text illustrates a typical background dynamic regime for our model. Here we also replot the psd of the LFP for this background regime on a log-log scale (see Fig 5).

Notice the following characteristics of the dynamical regimes we propose as candidates for exhibiting realistic background activity: First, the durations of the patterns are quite random and sometimes ill-defined, but they have a characteristic persistent time $\tau^{\text{persist}}_{\text{bkgrnd}}$ which can be considerably longer than τ_{slow}; this is consistent with several experimental observations [35, 108, 109]. In the regime shown, $\tau^{\text{persist}}_{\text{bkgrnd}} \sim 300$ ms, compared with $\tau_{\text{slow}} = 128$ ms. It is not hard to make $\tau^{\text{persist}}_{\text{bkgrnd}}$ last as long as 1-2 s by increasing L^E and decreasing the local couplings

One may be concerned that $\tau^{\text{persist}}_{\text{bkgrnd}}$ may increase with system size, and therefore $\tau^{\text{persist}}_{\text{bkgrnd}}$ may become unphysically large if the cluster size in our model were increased to match that of the real V1. This is a legitimate concern, as such increases in switching times are predicted in a number of theoretical studies [110, 112, 113]. In our model, however, there are several parameters that can be tuned to control the values of $\tau^{\text{persist}}_{\text{bkgrnd}}$. We demonstrate below (section 4.3) that by tuning these parameters, switching times can be made longer or shorter as desired for clusters 4 times the size of those used in Fig 2 of the main text.

Also notice that, following the onset of each pattern, activity among E-cells in the orientation domain in question continues well past the point when their inhibitory counterparts have been fully activated. This characteristic, which is very much in evidence in panel [A] and also reflected in the shapes of the cross-covariance curves in panel [B], is consistent with experimental measurements [39, 40].

One reason we draw attention to the two characteristics in the last paragraph is that they are at odds with [111], which previously proposed a different network regime also exhibiting correlated background activity. Unlike ours, their regime is highly inhibition-driven. The model network described in Section 1 is, in fact, flexible enough that it can also support the scenario described in [111] – for a different set of parameters than the ones proposed here, of course. We believe the scenario presented in this subsection fits better with experimental data.

4.2 Constraints on network parameters

The phenomenon associated with the background regimes discussed above places quite a strong constraint on the parameters of our model. The emergence of patterns hinges on the ability of the E-population to nucleate MFEs (which however must not involve too large a fraction of the cluster since such synchronous firing is not typically observed in functional regimes of real V1). That in turn depends on a number of factors, notably the strengths of the background drives S^Q_{drive} and η^{bkg}_Q (see Section 1.2), and the local couplings $S^{QQ'}_{\text{fast}}$.

Other things being equal, the closer to threshold a system operates, the more readily it generates MFEs. We have chosen the background drive in such a way the system is operating quite close to threshold. Long-range fast excitation L^E_{fast} must not be too small as it is responsible for recruiting clusters in multiple hypercolumns. Once recruitment takes place, $\tau^{\text{bkgrnd}}_{\text{persist}}$ depends strongly on the ratio of L^I_{slow} to L^E_{slow} (S^E_{slow} and S^I_{slow} are fixed for definiteness). If L^I_{slow} is too small and L^E_{slow} is too large, then an orientation which has recruited will dominate nearly indefinitely (i.e., $\tau^{\text{bkgrnd}}_{\text{persist}} \to \infty$). In general, for the regimes we have investigated, a larger ratio of L^I/L^E leads to a more rapid interference with the E-dominated MFEs, hence a shorter $\tau^{\text{bkgrnd}}_{\text{persist}}$. Finally, $S^{QQ'}_{\text{fast}}$ and the local connectivity across clusters have the greatest influence on non-iso-oriented exclusion, which also affects $\tau^{\text{bkgrnd}}_{\text{persist}}$ and the patterned activity.

It is obviously impossible to balance all of the relationships in the last paragraph 'by hand'. To locate the relevant parameter regions, we performed many parameter sweeps, using the results of simulations to help

identify parameters with desired properties. See Fig 6, which tracks the same two panels shown in Fig 1.

4.3 Background patterns in larger systems

The switching time $\tau_{\text{persist}}^{\text{bkgrnd}}$ of the regime exhibiting background patterns shown in section 4 does not necessarily increase with the system size. If the sizes N_E, N_I of the clusters in our model are increased, then the network parameters can be retuned slightly to maintain qualitatively similar background fluctuations with a comparable switching timescale $\tau_{\text{persist}}^{\text{bkgrnd}}$. In Fig 10A we show background patterns obtained from a system very similar to the one shown in Fig 2 of the main text, except that N_E and N_I are increased to 512 and the coupling parameters have been retuned slightly. As can be seen in Fig 10A, $\tau_{\text{persist}}^{\text{bkgrnd}}$ for this regime is ~ 300ms. By taking this regime and modifying the local coupling strengths $S^{IE} + S^{EI}$ and S^{EE} by $\pm 50\%$, as done in Fig 6, we can obtain qualitatively different regimes for which $\tau_{\text{persist}}^{\text{bkgrnd}}$ can be larger or smaller than 300ms. Examples of these other regimes are shown in Fig 10B,C.

5 Surround suppression of inhibitory and excitatory firing-rates

It has been observed that the firing rate of many excitatory cortical cells is lower when stimulated with an optimally-oriented full-field grating than with an optimally-oriented small grating subtending only the neuron's classical receptive field [31, 42, 43, 117, 118]. Typical theories regarding this type of effect invoke long-range or lateral connectivity (or feedback), as well as a strong inhibitory agent which is triggered by the activation of the surrounding hypercolumns, and which serves to suppress excitation within the central/measured hypercolumn [34, 119]. As there is no strong evidence for long-range inhibition within the cortex, the typical assumption is that the inhibitory mechanism leading to surround suppression must involve long-range excitation of the local inhibitory circuit. As such, many theories regarding surround suppression predict that, as the surround is stimulated, local inhibitory firing rates in the center would increase, thus suppressing excitatory firing-rates in the center.

Recent results of Ozeki et al. [42, 120] challenge the view expressed at the end of the last paragraph: their intracellular measurements infer that, when the surround and center are both stimulated with an optimally-oriented grating, both inhibitory and excitatory firing rates are lower than the corresponding rates when only the center is stimulated.

We mention also another set of results, by Haider et al. [43], which finds, using images that are rich in spatio-temporal structure, an increase in the firing-rate of fast-spiking interneurons when one stimulates the surround in addition to the center.

In this section we will focus primarily on iso-oriented surround suppression a la [42]. A few related results including contrast dependence and other kinds of stimuli are discussed in section 5.2.

5.1 Network regimes exhibiting surround-suppression a la [42]

We fix in our network an orientation j and a hypercolumn k. It will be assumed throughout that all stimulations take the form of drifting gratings with orientation j. We view the kth hypercolumn as the 'center', and any subset of the other hypercolumns as the 'surround'. (Recall from Section 1 that connectivity between all pairs of hypercolumns are statistically identical in our model.) We will compare the firing rates of excitatory and inhibitory neurons in the center, i.e. in the cluster C_{jk}, in the following two scenarios: In Scenario 1, we stimulate C_{jk} alone; in Scenario 2, we stimulate some subcollection of $\{C_{jk'}, \forall\ k'\}$ including C_{jk}; for simplicity, we denote the latter by $\cup_{k'} C_{jk'}$.

Following Ozeki et al. [42], we say a regime exhibits surround-suppression if, under strong stimulation, there is decreased firing for both excitatory and inhibitory neurons in C_{jk} as one goes from Scenario 1 to Scenario 2. All stimuli considered in section 5.1 are assumed to have high contrast.

We will demonstrate that there are parameter regions for which the system exhibits surround-suppression in the sense above. A sample of results is shown in Table 2, and a more detailed illustration of this phenomenon is presented in Fig 5 in the main text.

	unstim	stim C_{jk}	stim $\cup_{k'} C_{jk'}$
$[E]_{jk}$	2.8	56	7.6
$[I]_{jk}$	6.6	63	39

Caption for Table 2. Here we present steady-state excitatory and inhibitory firing-rates for a typical cluster C_{jk} in our model, recorded under a variety of stimulus paradigms. 'Unstim' means no cluster is stimulated. 'Stim C_{jk}' means C_{jk} is the only cluster stimulated, and 'Stim $\cup_{k'} C_{jk'}$' means that several clusters of the same orientation (including C_{jk}) are stimulated.

Fig 7 shows two panels analogous to those in Fig 6, illustrating the parameter constraints placed on the network with regard to surround-suppression a la [42]. One sees from Fig 7 that, in each of the panels, the intersection of the grey region with the domain bounded by the solid black curve is nonempty. *This is the set of parameters which simultaneously satisfy all requirements in the studies reported in the main text of this paper.* To get a rough idea of the size of this viable parameter region, see Fig 7, in which the highlighted square region shows a two-fold variation in parameters.

5.2 Related results

5.2.1 Contrast dependence

We report here on some very preliminary results regarding the response to stimuli of low- versus high-contrast, for optimally oriented drifting gratings of increasing diameters. We think of the constant $C_{E,k,j}$ in our external input (see Sect. 1.2) as varying from 0 to 1; with 0.125 corresponding to low and 0.5 to high contrast; these numbers are chosen to elicit roughly desirable firing rates as seen in real V1. Since there is no notion of geometric distance among hypercolumns in our model, we represent the 'diameter' of a stimulus by the total number of clusters stimulated. That is to say, we fix an orientation j, think of C_{j1} as the 'center' or cluster 1, and study the firing rates in cluster 1 when clusters 1 through n, i.e., $C_{j1}, \ldots, C_{jn}$ are stimulated. Plots of E-firing rates as functions of n for a few different contrasts are shown in Fig. 3A in the main text. These results show some resemblance to the experimental data in [41].

The effects of a high contrast stimulation are discussed in previous sections. For very low contrast, one may rationalize the small increase in firing rate in cluster 1 as n goes from 1 to 2 as follows: For a system that operates very close to threshold but in a low firing regime (such as in background or when very weakly stimulated), there is a tendency to nucleate MFEs when any amount of additional excitation is received, and MFEs lead to the formation and reinforcement of a theta-pattern, which leads to increased firing rates. On the other hand, long-range effects are, on average, suppressive. It appears that the tendency to form MFEs outweighs this suppressive effect for a system in a low enough firing regime, but the scale is tipped (quickly) as one raises its level of excitation. That would explain the initial increase in firing rate, as well as the decrease for $n > 2$ for the regime shown in Fig 3A in the main text. This thinking is corroborated by simulations.

5.2.2 Regimes in which I-firing increases with grating diameter

For nearly all reasonable parameters (not necessarily close to the patch considered in this paper) that we have investigated, excitatory firing rates at the center drop very definitively as we stimulate the surround in addition to the center. This, however, is not true for inhibitory firing rates, which can go in either direction. That is to say, there exist sizable regions in parameters away from the patch we consider on which our model exhibits behavior opposite to that described in Sect. 5.1 with respect to inhibitory firing. One way to get into such a regime is to start from our reference parameters, and alter the coupling strengths $S^{EI} + S^{EI}$ as well as the input η_I^{bkg} in order to decrease the relative fraction of I-firing that is triggered by MFEs. The fact that our model supports such a range of behaviors is testimony to its versatility; it also points to the importance of constraining parameters by experimental data and the need for further work.

5.2.3 Stimulus type influences surround-suppression

Our model is not capable of fully capturing the results of Haider et al. in part because their work studied fast-spiking interneurons, which are not well modeled by the integrate-and-fire neurons we use. More importantly,

the stimulus used in Haider et al. is in the form of a 'natural movie', with broadband spatio-temporal structures, and not a drifting grating with a fixed orientation (which is the only type of stimulus used in all of our results so far). However, for the same set of parameters used to generate the inhibitory surround-suppression shown in Fig 5 of the main text, we have seen that a full-field stimulus that affects multiple orientations produces significantly lower firing rates in the center compared to stimuli of comparable strengths directed at a single orientation. This has motivated us to crudely approximate the 'natural' stimuli of the kind used by Haider by a drive of the form:

$$\eta^{\text{stim}}_{Q,j,k,l}\left(t\right) = C_{Q,j,k}\eta^{\text{bkg}}_{Q}\left(1+\sin\left(\theta_j+\omega\left(t-\phi_l\right)\right)\right),$$

where j varies across all angles, and k varies across the hypercolumns directly affected by the stimulus. Using this 'natural' stimulus, we have found that our reference parameter-set (shown in Fig 5 of the main text) produces a rather different response; the inhibitory cells show a slight surround facilitation, whereas the excitatory cells remain suppressed. These observations are consistent with, respectively, the fast-spiking interneurons and regular-spiking pyramidal cells observed by [43]. An example of this is shown in Fig 8.

6 Comparison with related works

A variety of theories have been proposed to explain background phenomena and surround suppression in V1. We compare and contrast our findings with a few previous results.

6.1 Rate models

We mention first some results involving firing-rate models which can explain, to varying degrees, spontaneous background activity within V1. Work by [110] applies linear stability analysis to a nonlinear firing-rate model to reveal marginally stable states. By suitably balancing a noisy background input, their system can exhibit a background regime which fluctuates between different marginal modes with biologically realistic timescales. Further work by [110, 112, 121, 122] has investigated linear firing-rate models which are designed to amplify weak inputs to produce transient impulse responses. Using background drives with the appropriate spatio-temporal correlations these models can also exhibit background-fluctuations with biologically realistic spatio-temporal scales.

An important difference between our results and [112] is that our regime does not rely on correlations in the drive to produce patterned activity. While background drive is likely correlated, there is evidence that these correlations may not be directly responsible for the patterns observed in V1 (see [108]). We also differ from [110] in that, though not the most common scenario, it is not rare for multiple orientations to be represented within the same sustained pattern. This phenomenon, present in our regime but excluded in [110], is consistent with [38]. In addition to these differences, the regimes produced by our model exhibit MFEs (see main text). This self-organized collaborative activity among neurons (i.e., the interplay of MFEs and the characteristic times between them) are not readily captured by current firing-rate or population-dynamics models. Because our network does not coarse-grain over individual firing-events, our model can capture the structure of correlated firing-events, both in terms of magnitude and distribution [39, 40]. These correlated firing-events may be important for coding [123–131] and they cannot be readily captured by firing-rate models.

6.2 Network Models

There are network-based models of V1 that are closer in spirit to our approach than the firing-rate models discussed above. One such network model is included in Murphy and Miller [112]; this model exhibits dynamics which are well captured by the firing-rate model discussed in the same paper. Another network model of V1 is Cai et al. [111]. This model, which was used earlier to investigate spontaneous background activity, has influenced some of the thinking in the present paper. The regimes that we regard as correctly reflecting experimental observations, however, differ substantially from those proposed in [111], and we would like to discuss that in some detail.

The most significant difference between the regime proposed in [111] and ours is that the former is largely inhibition-dominated, while the regime proposed here is balanced in a way that permits serious sustained

competition between its excitatory and inhibitory populations; in that sense, our regime is poised in a position of much higher gain. These differences manifest themselves clearly in the firing patterns in background: In [111], the initiation of a pattern, called a 'recruitment event', takes the form of a single burst of excitatory firing. Following a recruitment, the regime invokes strong slow-excitation to sustain θ-specific inhibitory firing. Further excitatory firing beyond that one initial burst is prohibited in these post-recruitment periods of θ-specific activity, which often conclude with the initiation of a new recruitment-event within a different θ. See Fig 9. As a result of this dynamic mechanism, the persistence timescale for background patterns in [111] is directly controlled by τ_{slow}, and can never be substantially longer than τ_{slow}. Another byproduct of its mostly-inhibitory firing in background is that this regime has unrealistically low firing rates both in background and under drive.

Our model is flexible enough that it has the capability to produce, in different parameter regions, both the regimes we propose and the scenario in [111]; for the latter, take L^I_{fast} or $L^I_{slow} >> L^E_{slow}$. We have a number of reasons, however, for believing that the regimes we propose in this paper more accurately reflect real V1 dynamics: (1) The parameter regions associated with the inhibitory-dominated regime similar to [111] do not exhibit biologically-realistic firing-rates, whereas our regimes do, as ensured by the benchmarking process in section 1.3; see Fig 1. (2) Sustained excitatory firing in background is supported by direct experimental data from [39]. (3) We know of no evidence to suggest that the time scale $\tau^{\text{bkgrnd}}_{\text{persist}}$ should be directly tied to τ_{slow}. On the contrary, spontaneous activity in eye-closed ferret V1 can last many seconds, which is longer than the persistence timescales of many (but not all) types of NMDA receptors [108], and certain types of spontaneous cortical activity persist with similar timescales even under NMDA blockade [115]. (4) Our fourth reason has to do with another V1 phenomenon, namely surround suppression, which we discussed in section 5. Due to their extreme inhibitory suppression, regimes we have found that exhibit background patterns similar to those in [111] exhibit classical surround-suppression of the excitatory population but not surround-suppression of the excitatory and inhibitory population as is observed in recent experiments [42].

For these reasons, we believe that the regimes presented in the main text of this paper are more realistic than the regimes presented in [111], and that the mechanisms discussed in this paper and in [95] are relevant to the functioning of V1.

7 Appendix

All of the parameters in our model are identified in Sects. 1.2 and 1.3. Many sweeps were conducted to locate regions in this rather high dimensional parameter space. Our search was semi-systematic: We began with some initial educated guesses. At the same time, we attempted to identify 'opposing forces' in the system that need to be balanced. Suppose X and Y form such a pair (examples of which are given below). We pitched them against one another in simulations of regimes corresponding to 2D panels in which both X and Y are varied and other parameters are guessed. Computation results were used to identify pairs (X, Y) that manifest the desired phenomena. Information on this 2D study (especially when it failed to produce reasonable parameters) gave feedback to our initial guesses, which were successively improved.

Via these parameter sweeps, we located an open region in parameter space with all of the desired properties. This region is reasonably sized: in most directions, one can vary the parameter in question by 40% and remain in the viable set. A specific set of parameters in the interior of this region is fixed and is used in many of the simulations shown. We will refer to this set of parameters as our 'reference set'; its precise values are given below. Our search is by no means exhaustive, and we do not claim that parameters in this region are the only viable ones. Among the choices that we made, the most important are parameters related to background drive: we set them to put our system in a 'high-gain' mode, so it responds sensitively to changes in input (see Sect. 1.3). Two sets of 'opposing forces' we have found to require the most delicate balancing are, not surprisingly,

(i) local fast excitation vs inhibition, i.e. the coupling strengths $S^{QQ'}$, and
(ii) long-range slow excitation to E vs I-neurons, i.e. the coupling strengths L^E vs L^I.

Other parameters are relatively stable, which is not to say that they can be arbitrarily fixed (e.g. too small a L^{EE}_{fast} will hamper the generation of MFEs discussed in the main text). They are relatively stable in the sense that once appropriately chosen, varying them by reasonable amounts will not lead to dramatic qualitative changes in the dynamics, though precise values of other parameters have to be adjusted.

With regard to (i), we set $S^{II} = 0$, our rationale being that a positive S^{II} amounts to weaker inhibition. To further simplify the situation, we chose to vary S^{EI} and S^{IE} together, viewing the combined value of these two parameters as a measure of the effectiveness of the inhibitory population. Thus in (i), we pitch S^{EE}, representing the effectiveness of the local fast excitation, against S^{EI} and S^{IE}, which are varied simultaneously. Among all of the 2D panels we have studied, this is quite possibly the one that placed the most serious constraints on the model. The panel L^E vs L^I also revealed important constraints, especially with regard to producing data that fit with experimental observations in unstimulated background activity (see Section 4). Constraints on parameters in these two 2D-slices are tracked in successive steps in Figs 1, 6 and 7.

Reference parameter set

The network size is given by: $N_I = N_E = 128$, $J = 3$, $K = 8$.

The feedforward input is governed by: $S^I_{\text{drive}} = 0.0134$, $S^E_{\text{drive}} = 0.01364$, $\eta^{\text{bkg}}_I = 0.2875$, $\eta^{\text{bkg}}_E = 0.5324$.

The local network architecture is determined by the local connectivity fractions $p^{QQ'}_{jjkk}$ between the presynaptic Q' and postsynaptic Q populations in a single orientation domain, as well as the analogous fraction and $p^{QQ'}_{jj'kk}$ between populations in different orientation domains within the same hypercolumn. These fractions are given by: and $p^{II}_{jjkk} = 1$, $p^{IE}_{jjkk} = 0.5$, $p^{EI}_{jjkk} = 1$, $p^{EE}_{jjkk} = 0.25$, $p^{II}_{jj'kk} = 0.75$, $p^{IE}_{jj'kk} = 0.375$, $p^{EI}_{jj'kk} = 0.75$, $p^{EE}_{jj'kk} = 0.0625$. The long-range network architecture is determined by the connectivity fractions $p^{QE}_{jjkk'}$ between the presynaptic E and postsynaptic Q populations in different orientation domains of equivalent orientation preference. These fractions are: $p^{QE}_{jjkk'} = 0.125$.

The connection strengths are given by: $S^{II}_{\text{fast}} = 0$, $S^{IE}_{\text{fast}} = 0.008175$, $S^{EI}_{\text{fast}} = 0.007619$, $S^{EE}_{\text{fast}} = 0.026593$, $S^{II}_{\text{slow}} = 0$, $S^{IE}_{\text{slow}} = 0.000313$, $S^{EI}_{\text{slow}} = 0$, $S^{EE}_{\text{slow}} = 0.002345$, $L^{II}_{\text{fast}} = 0$, $L^{IE}_{\text{fast}} = 0.006250$, $L^{EI}_{\text{fast}} = 0$, $L^{EE}_{\text{fast}} = 0.003910$, $L^{II}_{\text{slow}} = 0$, $L^{IE}_{\text{slow}} = 0.008281$, $L^{EI}_{\text{slow}} = 0$, $L^{EE}_{\text{slow}} = 0.000938$.

The long-range time-constant is given by $\tau^E_{\text{slow}} = 128$ms.

parameter set for larger models used in section 4.3 The local connectivity fractions p^{II}_{jjkk}, p^{EI}_{jjkk}, $p^{II}_{jj'kk}$, and $p^{EI}_{jj'kk}$ have been modified to 0.75, 0.75, 0.42 and 0.42, respectively. The connection strengths have also been modified slightly, and are given by: $S^{II}_{\text{fast}} = 0.00217$, $S^{IE}_{\text{fast}} = 0.003153$, $S^{EI}_{\text{fast}} = 0.004621$, $S^{EE}_{\text{fast}} = 0.009366$, $S^{II}_{\text{slow}} = 0$, $S^{IE}_{\text{slow}} = 0.000078$, $S^{EI}_{\text{slow}} = 0$, $S^{EE}_{\text{slow}} = 0.000584$, $L^{II}_{\text{fast}} = 0$, $L^{IE}_{\text{fast}} = 0.001563$, $L^{EI}_{\text{fast}} = 0$, $L^{EE}_{\text{fast}} = 0.000977$, $L^{II}_{\text{slow}} = 0$, $L^{IE}_{\text{slow}} = 0.001464$, $L^{EI}_{\text{slow}} = 0$, $L^{EE}_{\text{slow}} = 0.000234$. The rest of the parameters are the same as those given in the reference parameter set above.

Figure 1:

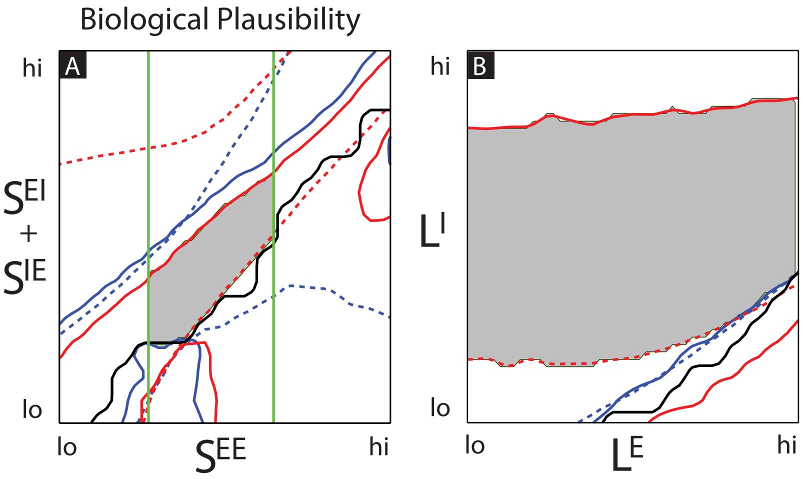

Caption for Fig 1: Illustration of parameter exclusion for biological plausibility. Viable values of parameters in two 2D-slices of parameter space passing through our 'reference set' are shown. In panel [A] we fix all parameters except for S^{EE}, S^{IE} and S^{EI}, varying S^{IE} and S^{EI} simultaneously (the combination of S^{IE} and S^{EI} represents the effectiveness of the inhibitory mechanism). The variation in parameters is performed with small multiplicative steps of size 2ˆ(1/4) so that the extreme endpoints differ by a factor of 16. In panel [B] we fix all parameters except for L^E and L^I, which we vary with the same multiplicative step sizes as in panel [A]. The central point in both of these panels corresponds to the reference set given in Supp. Mat. A. We consider here the steady state firing rates of our system in background (denoted by m_Q^{bkg}), and under drive (m_Q^{stim}). Within both panels [A] and [B], the dashed red curves bound a parameter region wherein 1Hz $< m_E^{\text{bkg}} <$ 5Hz. The dashed blue curves bound a region wherein 3Hz $< m_I^{\text{bkg}} <$ 15Hz. The solid red curves bound the region 20Hz $< m_E^{\text{stim}} <$ 75Hz. The solid blue curves bound the region 20Hz $< m_I^{\text{stim}} <$ 75Hz. To the right of the black curve the system exhibits frequent strong synchronous firing-events, which we regard as unbiological. Finally, the two vertical green lines within panel [A] indicate the boundaries of the region where the fast local EPSPs lie within a biologically plausible range (i.e, 0.5 $\rightarrow$ 1.5mV). Thus, the parameters in the grey region are the only ones that satisfy all of the constraints above, and we will henceforth consider only parameters in this region.

Figure 2:

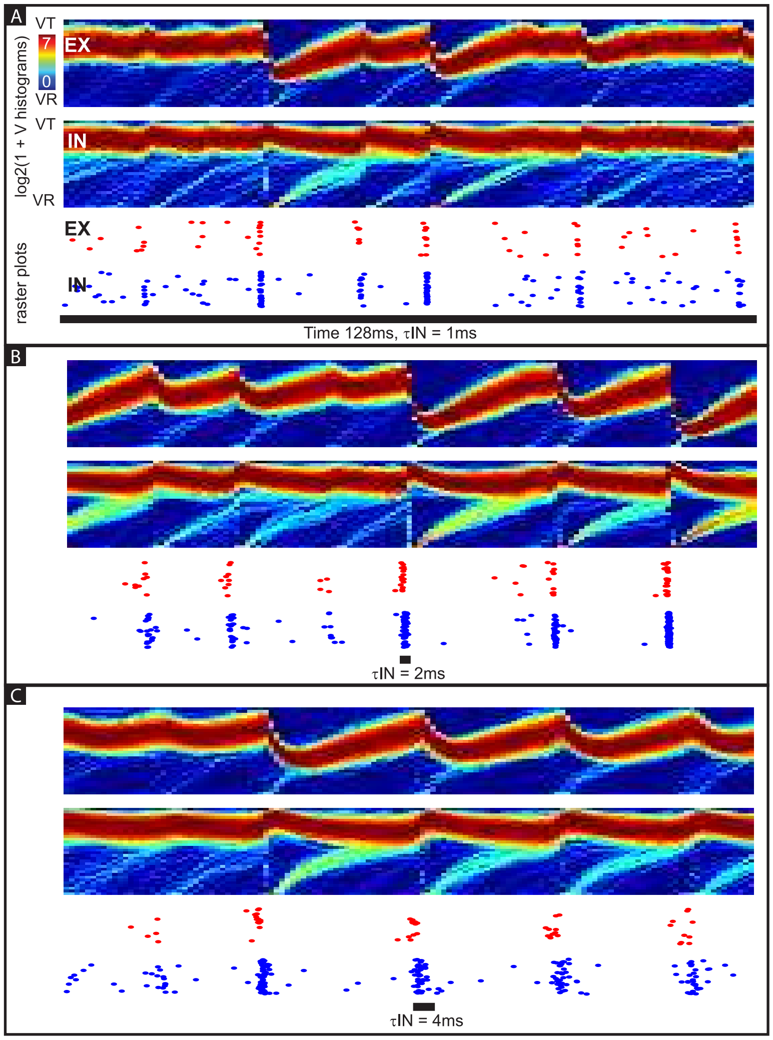

Caption for Fig 2:

Each panel shows typical trajectories for one of a family of networks. Each network in this family is a single cluster of $N_E = 1024$ excitatory neurons and $N_I = 1024$ inhibitory neurons driven by a uniform Poisson background input. Each neuron is modeled using the conductance-based integrate-and-fire equations with a nonzero τ_E, τ_I. The ratio of τ_I/τ_E is fixed to be 2 in accordance with physiologically observed timescales for GABA-type inhibition and AMPA-type excitation (the precise ratio here is not critical to our results). The different networks are identical except for the value of τ_I, which ranges from 1ms (in panel-A) to 4ms (in panel-C). In the top two strips of each panel we show the voltage-distributions for the neurons in the network. Each vertical strip represents the voltage histogram for the EX or IN neurons in the network at a particular time within a 128ms-long trajectory. The bottom two strips in each panel show the raster plots for the EX and IN neurons in the network. Note that the presence of MFEs is very apparent, even when τ_I is large. Moreover, the width of the MFEs is $\leq$ 10ms, and the larger MFEs are clearly distinguishable.

Figure 3:

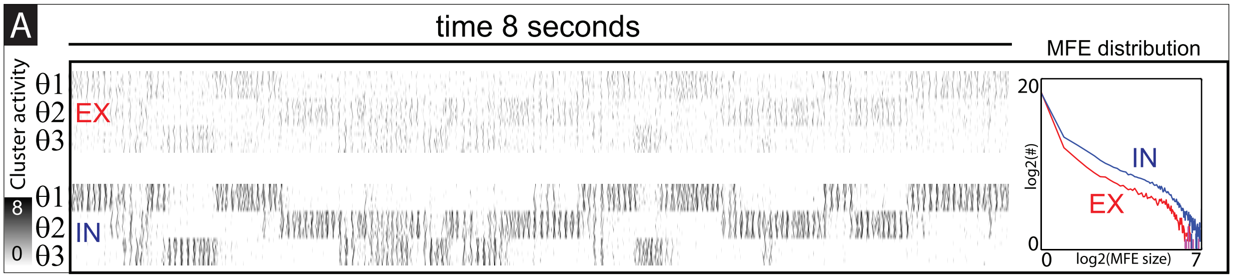

Caption for Fig 3: On the left we show a panel illustrating a typical trajectory in background for a network with a long-range transmission delay of 9ms between hypercolumns. Other than the addition of these delays, the network architecture is identical to the model discussed in the main text. The coupling strengths have been altered by ±10% to preserve the dynamical regime. On the right we plot the MFE distribution for this regime.

Figure 4:

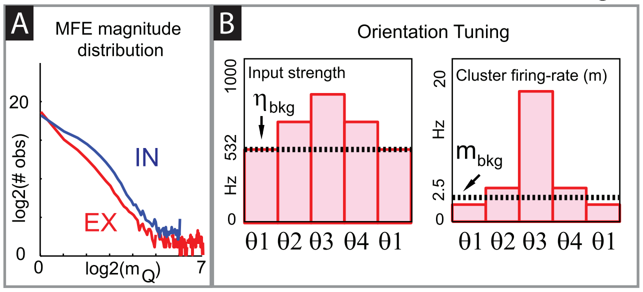

Caption for Fig 4: Illustration of orientation sharpening. Here we construct a model which resolves orientation space more finely (i.e., with $J = 4$). We retune this model so that the dynamic regime is qualitatively similar to the $J = 3$ model discussed in the main text. [A]. MFE distribution. [B]. Illustration of orientation tuning for this model. On the left we present the broadly tuned feedforward input we use to stimulate the model (rates shown for excitatory neurons). We measure this strength in terms of the time-averaged Poisson input rate associated with a typical neuron of a given orientation within the stimulated hypercolumns. The background rate is indicated with a dashed line. On the right we plot the time-averaged output excitatory firing-rates for the stimulated hypercolumns. The background firing-rate is indicated with a dashed line. Note that the output firing-rates are much more sharply tuned than the input firing-rates.

Figure 5:

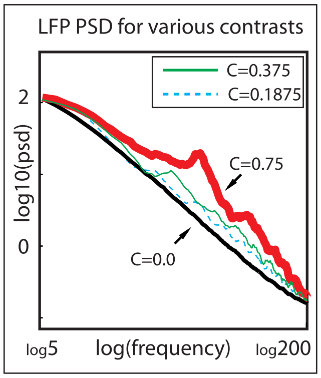

Caption for Fig 5: Power spectral density (averaged over nonoverlapping 512ms windows) of the population averaged voltage for a single cluster in our model both under background (black) and under low-contrast (green) and high-contrast drive (red). Note that the frequency axis is plotted on a logarithmic scale.

Figure 6:

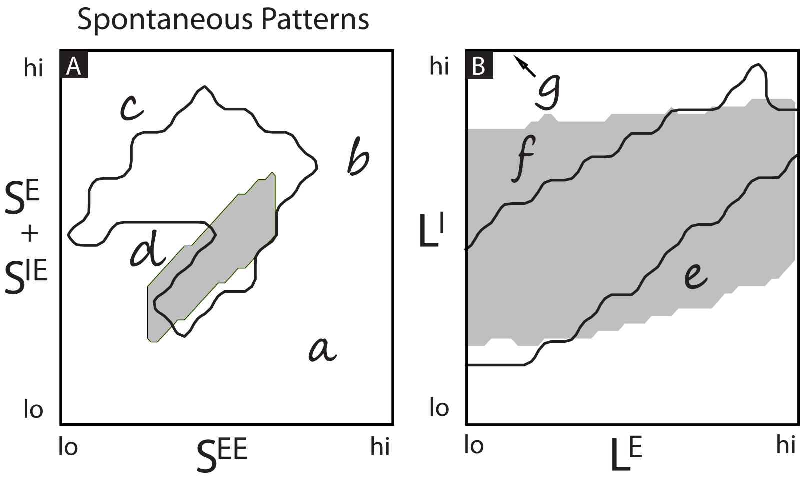

Caption for Fig 6: Illustration of parameter exclusion for background activity. Panels [A] and [B] show admissible parameters in the corresponding 2D-slices shown in Fig 1. In each of these panels, the grey region shown is a (large) subset of the grey region in Fig 1, with some additional parameters excluded due to inadequate performance with respect to orientation selectivity and/or receptive fields (see Sect. 3). Parameters in the region bounded by the black curves are admissible for purposes of background activity. In panel [A], parameters near 'a' are excluded due to too much synchronous firing (S^{EE} too large relative to S^{IE}, S^{EI}). Parameters near 'b' are excluded because heightened activity in one orientation domain tends to spill over into other orientation domains (local couplings too strong). Parameters near 'c' are excluded because these regimes show no discernible patterns in background (strong inhibition prevents successful recruitment and dominance by any one θ). Parameters near 'd' are excluded because the patterns switch too slowly (insufficient inhibition to suppress E-activity once it is aroused). In panel [B], patterns switch too slowly for parameters next to 'e', too fast for parameters near 'f'. In the region above 'g' (i.e. larger L^I still), the regime becomes increasingly inhibition driven, showing characteristics of the regimes proposed in [111].

Figure 7:

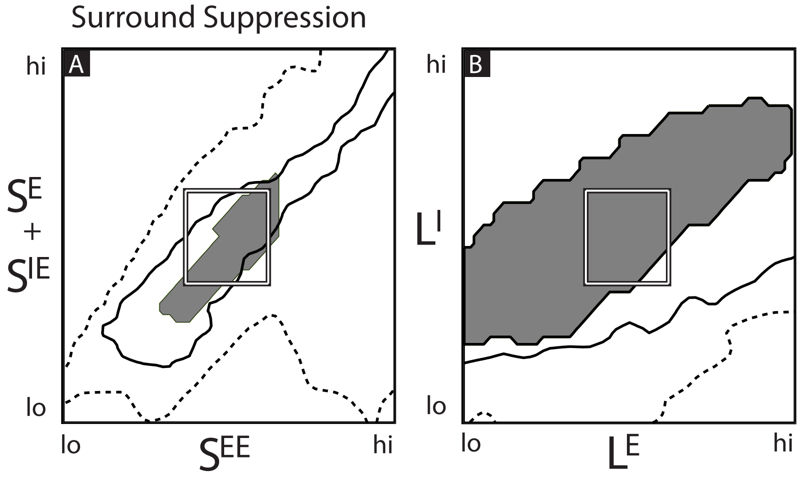

Caption for Fig 7: Parameter constraints imposed to satisfy surround suppression. The 2D-slices of parameter space shown here are identical to those in Figs 1 and 6, with identical labeling of axes. In each panel, the grey region is obtained by intersecting the corresponding grey region in Fig 6 with the region bounded by the black curves there, that is to say, parameters within the grey regions shown here meet all previous conditions. Fig 7 pertains to the effects of strong stimulation. Requiring that excitatory firing rate is lowered in the center as one stimulates the surround imposes no further constraint on parameters. In each of the panels here, the region bounded by the solid black curve consist of parameters with the property that when strongly stimulated, inhibitory firing is lower by $\geq 45\%$ in Scenario 2 compared to Scenario 1, and the dashed black curve bounds the region where inhibitory firing in Scenario 2 is lower than or equal to that in Scenario 1. Note that the parameter region which satisfies all of our constraints is not small – the highlighted square region indicates a two-fold variation in parameters.

Figure 8:

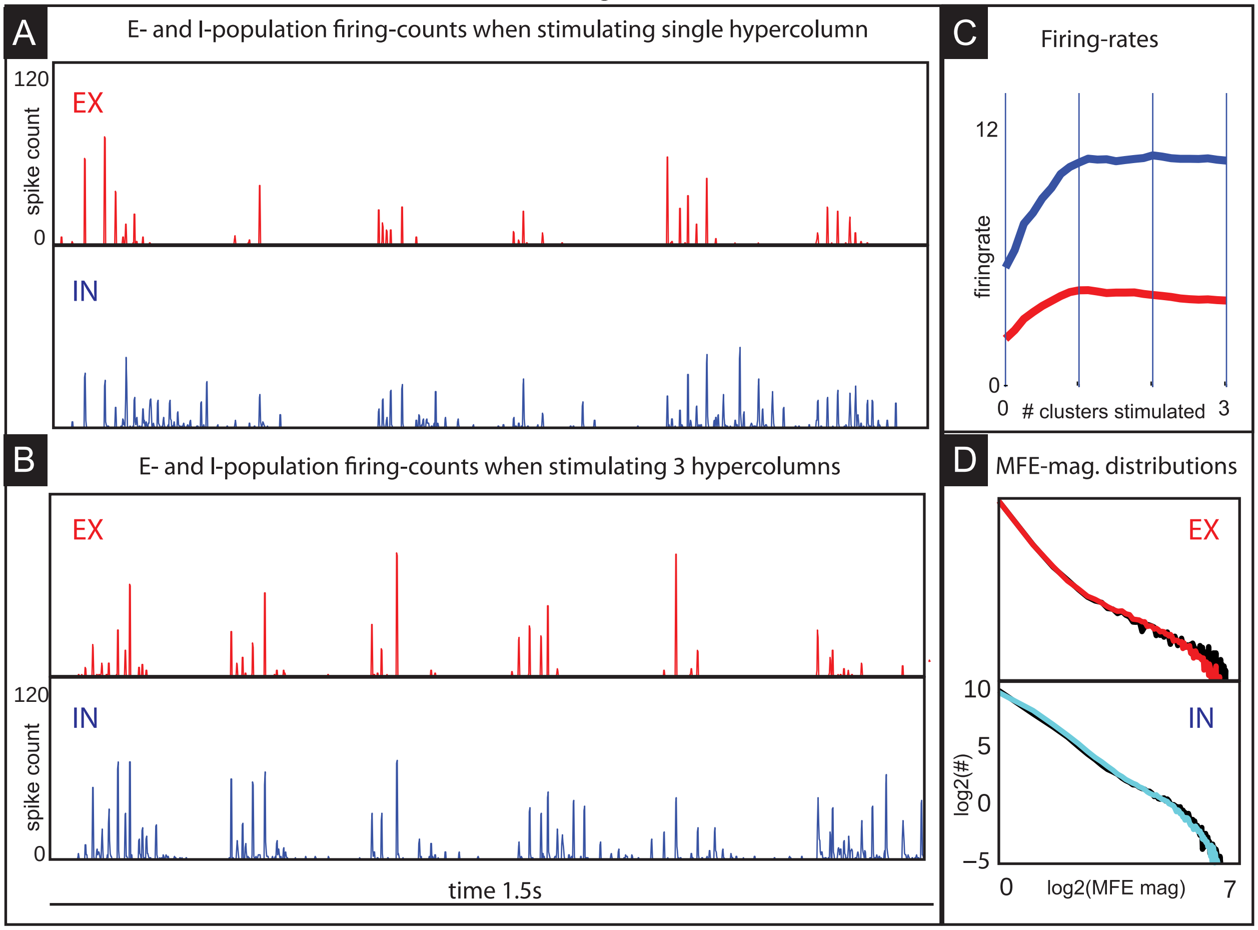

Caption for Fig 8: In panels A and B we show the firing-events produced by our reference parameter set under the 'natural' drive described in section 5.2.3. Panel-A illustrates the sequence of E- and I-population spike counts (collected over 1ms bins) produced by one of the clusters C_{jk} in our network when hypercolumn k alone is stimulated. Panel-B illustrates these spike-counts when 2 other hypercolumns are also stimulated. In Panel-C we show time-averaged firing-rates for the E- (red) and I- (blue) populations as a function of the number of clusters stimulated. We did not divide the I-populations into MFE-spikes and nonMFE-spikes (as done in Fig 5 of the main text), since the firing-rates were quite low and the '95%' rule was not a good qualitative assessment of the degree of collaborative activity in this regime. In Panel-D we show the histograms of MFE magnitudes for E- (top) and I-cells (bottom) for the regimes shown in panels A (black) and B (colored) respectively.

Figure 9:

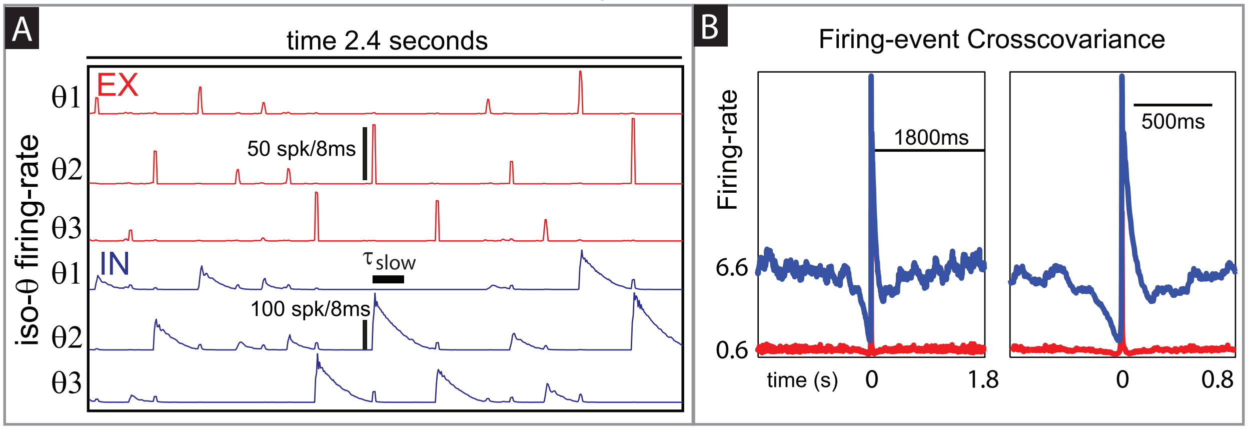

Caption for Fig 9: Panel [A]: Here we show 2.4s of dynamics from a typical background regime near region 'g' in Fig 6, with a ratio of $L^I/L^E = 200$. This regime produces rasters and traces similar to those in [111]. The format follows that of panel [A] in Fig 2 of the main text, with two scale bars for the excitatory and inhibitory populations. Note that the dynamics in this regime are strongly stereotyped: sharp excitatory MFEs (called recruitments in [111]) initiate inhibitory-dominated epochs of firing which preclude further excitatory firing — in contrast to the sustained competition between E and I in our model following the initiation of each pattern. Also, as the inhibitory epochs in [111] are driven by g_{slow}, the inhibitory firing-rate during these epochs follows a predictable decay. Panel [B]: (i) cross-covariances, as defined in the methods section of the main text, for $Q = E$ (red) and $Q = I$ (blue) over 1800ms (left) and $\sim$800ms (right); note the strong time-asymmetry at time 0. Finally, note the sharp contrast between the cross-covariances exhibited by this inhibitory dominated regime and the cross-covariances exhibited by the regime presented in Fig 6.

Figure 10:

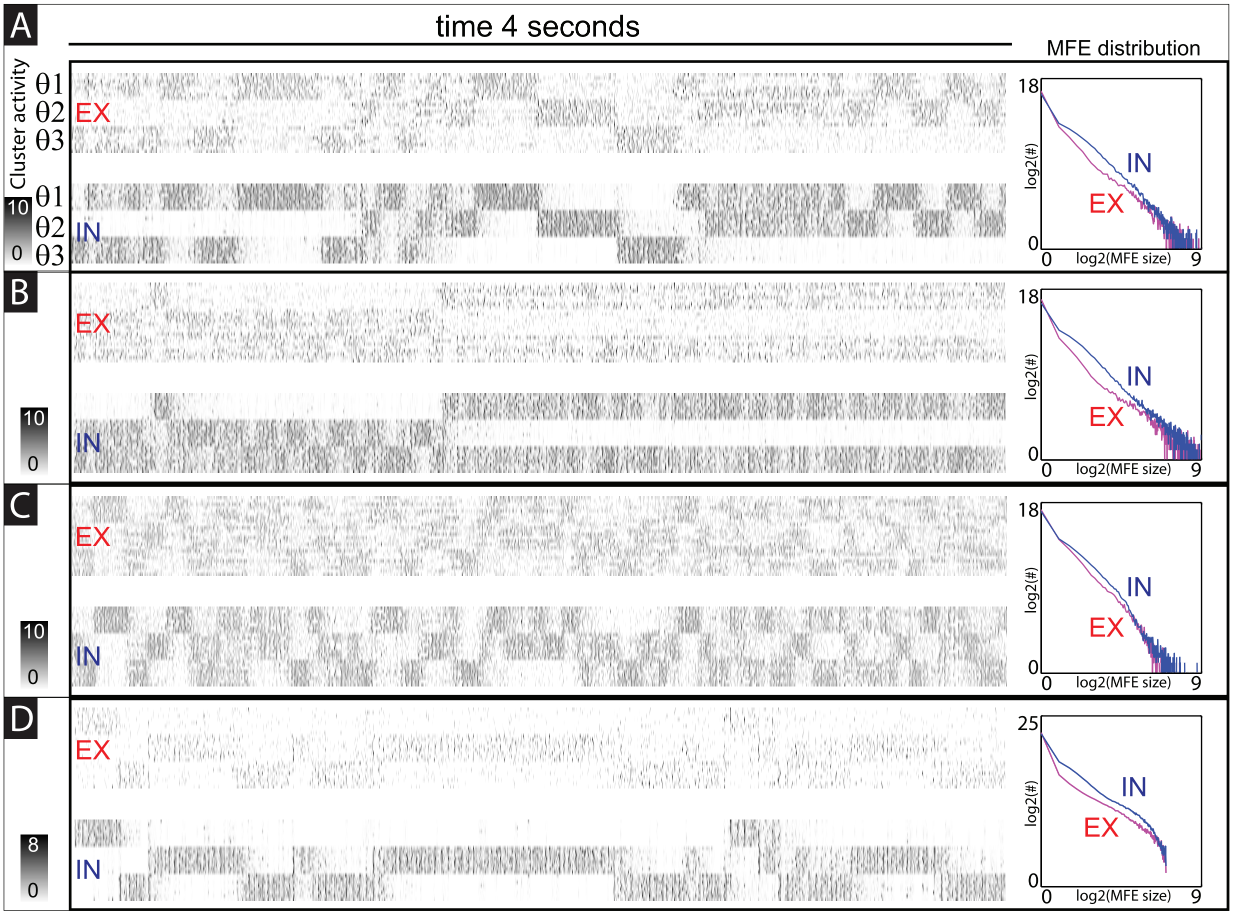

Caption for Fig 10:

In each panel we present a dynamical regime exhibiting background activity. The format of these panels is similar to that of Fig 2 in the main text. Each panel shows a raster-plot of cluster activity on the left, and a log-log plot of the distribution of MFE sizes on the right. In panels A,B,C the size of each cluster is $N_E, N_I = 512$; the parameters for panel-A are described in Supp. Mat. B. Note that the switching time in panel-A is on the same order as that shown in Fig 2 of the main text (part of which is reproduced in panel-D of this figure). In panel-B we show background activity for a system equivalent to that shown in panel-A, except that the local coupling strengths $S^{IE} + S^{EI}$ have been decreased by $\sim 20\%$ and S^{EE} has been increased by $\sim 20\%$. For this regime the typical MFEs are slightly larger than in panel-A (as can be seen by inspecting the tail end of the MFE distribution) and the switching time is longer. In panel-C we show background activity for another system equivalent to that shown in panel-A, except that the local coupling strengths have been modified in the opposite direction – S^{EE} has been decreased and $S^{IE} + S^{EI}$ have been increased. For this regime the typical MFEs are slightly smaller than in panel-A, and the switching time is shorter. In panel-D we reproduce part of the dynamic trace shown in Fig 2 of the main text, which corresponds to a system with cluster size $N_E = N_I = 128$.

References

[1] K. Tanaka. Organization of geniculate inputs to visual cortical cells in the cat. *Vis. Res.*, 25:357–364, 1985.

[2] C. D. Gilbert and T.N. Wiesel. Columnar specificity of intrinsic horizontal connections and corticocortical connections in cat visual cortex. *J. Neurosci.*, 9:2432–442, 1989.

[3] B.A. McGuire, C.D. Gilbert, P.K. Rivlin, and T.N. Wiesel. Targets of horizontal connections in macaque primary visual cortex. *J. Comp. Neurol.*, 305:370–392, 1991.

[4] J. Hirsch and C.D. Gilbert. Synaptic physiology of horizontal connections in the cat's visual cortex. *J. Neurosci.*, 11:1800–1809, 1991.

[5] A. Peters and E. Yilmaz. Neuronal organization in area 17 of cat visual cortex. *Cereb. Cortex*, 3:49–68, 1993.

[6] L.P. O'Keefe, J.B. Levitt, D.C. Kiper, R.M. Shapley, and J.A. Movshon. Functional organization of owl monkey lateral geniculate nucleus and visual cortex. *J. Neurophysiol.*, 80:594–609, 1998.

[7] E. Callaway. Local circuits in primary visual cortex of the macaque monkey. *Ann. Rev. Neurosci.*, 21:47–74, 1998.

[8] G. DeAngelis, R. Ghose, I. Ohzawa, and R. Freeman. Functional micro-organization of primary visual cortex: Receptive field analysis of nearby neurons. *J. of Neurosci.*, 19:4046–4064, 1999.

[9] Y. Yoshimura, H. Sato, K. Imamura, and Y. Watanabe. Properties of horizontal and vertical inputs to pyramidal cells in the superficial layers of the cat visual cortex. *J Neurosci*, 20:1931–1940, 2000.

[10] J.A. Hirsch. Synaptic physiology and receptive field structure in the early visual pathway of the cat. *Cereb. Cortex*, 13:63–69, 2003.

[11] D. Hubel and T. Wiesel. Laminar and columnar distribution of geniculo cortical fibers in the macaque monkey. *J Comp Neurol*, 146:421–450, 1972.

[12] R.T. Marrocco, J.W. McClurkin, and R.A. Young. Modulation of lateral geniculate nucleus cell responsiveness by visual activation of the corticogeniculate pathway. *J. Neurosci.*, 2:256–263, 1982.

[13] E. Kaplan and R. Shapley. X and Y cells in the lateral geniculate nucleus of the macaque monkey. *J.Physiol.*, 330:125–143, 1982.

[14] T.J. Gawne, J.W. McClurkin, B.J. Richmond, and L.M. Optican. Lateral geniculate neurons in behaving primates. iii. response predictions of a channel model with multiple spatial-to-temporal filters. *J. Neurophysiology*, 66:809–823, 1991.

[15] J.W. McClurkin, T.J. Gawne, L.M. Optican, and B.J. Richmond. Lateral geniculate neurons in behaving primates. ii. encoding of visual information in the temporal shape of the response. *J. Neurophysiology*, 66:794–808, 1991.

[16] C.D. Gilbert. Horizontal integration and cortical dynamics. *Nature*, 9:1–13, 1992.

[17] A.M. Sillito, J. Cudeiro, and P.C. Murphy. Orientation sensitive elements in the corticofugal influence on centre- surround interactions in the dorsal lateral geniculate nucleus. *Exp Brain Res*, 93:6–16, 1993.

[18] A. Grinvald, E.E. Lieke, R.D. Frostig, and R. Hildesheim R. Cortical point-spread function and long-range lateral interactions revealed by real-time optical imaging of macaque monkey primary visual cortex. *J. Neurosci.*, 14:2545–2568, 1994.

[19] R.C. Reid and J.-M. Alonso. Specificity of monosynaptic connections from thalamus to visual cortex. *Nature*, 378:281–284, 1995.

[20] J. Cudeiro and A.M. Sillito. Spatial frequency tuning of orientation-discontinuity-sensitive corticofugal feedback to the cat lateral geniculate nucleus. *J Physiol*, 490:481–492, 1996.

[21] D.W. Godwin, S.C. Van Horn, A. Eriir, M Sesma, C. Romano, and S.M. Sherman. Ultrastructural localization suggests that retinal and cortical inputs access different metabotropic glutamate receptors in the lateral geniculate nucleus. *J. Neurosci.*, 16:8181–8192, 1996.

[22] W.H. Bosking, Y. Zhang, B. Schofield, and D. Fitzpatrick. Orientation selectivity and the arrangement of horizontal connections in tree shrew striate cortex. *J. Neurosci.*, 17:2112–2127, 1997.

[23] H. Asi, J. Lund, G. Blasdel, A. Angelucci, and J. Levitt. Stripe like patterns of lateral connections in layers 4B and upper 4C of macaque monkey primary visual cortex area V1. *Preprint*, 1998.

[24] N.H. Yabuta and E.M. Callaway. Cytochrome oxidase blobs and intrinsic horizontal connections of layer 2/3 pyramidal neurons in primate V1. *Visual Neurosci.*, 15:1007–1027, 1998.

[25] Y. Dan, J.M. Alonso, W.M. Usrey, and R.C. Reid. Coding of visual information by precisely correlated spikes in the lateral geniculate nucleus. *Nat. Neurosci.*, 1:501–507, 1998.

[26] P. Murphy, S. Duckett, and A. Sillito. Feedback connections to the lateral geniculate nucleus and cortical response properties. *Science*, 286:1552–1554, 1999.

[27] V. Bringuier, F. Chavane, L. Glaeser, and Y. Fregnac. Horizontal propagation of visual activity in the synaptic integration field of area 17 neurons. *Science*, 283:695–699, 1999.

[28] C. Gutierrez, C.L. Cox, J. Rinzel, and S.M. Sherman SM. Dynamics of low-threshold spike activation in relay neurons of the cat lateral geniculate nucleus. *J. Neurosci.*, 21:1022–1032, 2001.

[29] J.-M. Alonso, W. Martin Usrey, and R. Reid. Rules of connectivity between geniculate cells and simple cells in cat primary visual cortex. *J. Neurosci.*, 21:4002–4015, 2001.

[30] H.J. Chisum, F. Mooser, and D. Fitzpatrick. Emergent properties of layer 2/3 neurons reflect the collinear arrangement of horizontal connections in tree shrew visual cortex. *J. Neurosci.*, 23:2947–960, 2003.

[31] W Bair, J.R. Cavanaugh, and J.A. Movshon. Time course and time-distance relationships for surround suppression in macaque v1 neurons. *J. Neurosci*, 23:7690–7701, 2003.

[32] J. Marino, J. Schummers, D.C. Lyon, L. Schwabe, O. Beck, P. Wiesing, K. Obermayer K, and M. Sur. Invariant computations in local cortical networks with balanced excitation and inhibition. *Nat. Neurosci.*, 8:194–201, 2005.

[33] A. Angelucci and P.C. Bressloff. Contribution of feedforward, lateral and feedback connections to the classical receptive field center and extra-classical receptive field surround of primate v1 neurons. *Progress in Brain Research*, 154:93–120, 2006.

[34] L. Schwabe, K. Obermayer, A. Angelucci, and P.C. Bressloff. The role of feedback in shaping the extra-classical receptive field of cortical neurons: A recurrent network model. *Journal of Neuroscience*, 26:9117–9129, 2006.

[35] M. Tsodyks, T. Kenet, A. Grinvald, and A. Arieli. Linking spontaneous activity of single cortical neurons and the underlying functional architecture. *Science*, 286:1943–1946, 1999.

[36] H. Spors and A. Grinvald. Spatio-temporal dynamics of odor representations in the mammalian olfactory bulb. *Neuron.*, 34:301–315, 2002.

[37] A. Grinvald, A. Arieli, M. Tsodyks, and T. Kenet. Neuronal assemblies: Single cortical neurons are obedient members of a huge orchestra. *Biopolymers*, 68:422–436, 2003.

[38] T. Kenet, D. Bibitchkov, M. Tsodyks, A. Grinvald, and A. Arieli. spontaneously emerging cortical representations of visual attributes. *Nature*, 425:954–956, 2003.

[39] A. Kohn and M.A. Smith. Stimulus dependence of neuronal correlation in primary visual cortex of the macaque. *J Neurosci.*, 25:3661–73, 2005.

[40] M.A. Smith and A. Kohn. Spatial and temporal scales of neuronal correlation in primary visual cortex. *J Neurosci.*, 28:12591–12603, 2008.

[41] M.P. Sceniak, D.L. Ringach, M.J. Hawken, and R. Shapley. Contrast's effect on spatial summation by macaque v1 neurons. *Nat. Neurosci.*, 2:733–739, 1999.

[42] H. Ozeki, I.M. Finn, E.S. Schaffer, K.D. Miller, and D. Ferster. Inhibitory stabilization of the cortical network. *Neuron*, 62:578–592, 2009.

[43] Bilal Haider, Matthew R. Krause, Alvaro Duque, Yuguo Yu, Jonathan Touryan, James A. Mazer, and David A. McCormick. Synaptic and network mechanisms of sparse and reliable visual cortical activity during nonclassical receptive field stimulation. *Neuron*, 65(1):107 – 121, 2010.

[44] W.R. Softky and C. Koch. The highly irregular firing of cortical cells is inconsistent with temporal integration of random epsps. *J. Neurosci.*, 13:334–350, 1993.

[45] C. Monier L. Borg-Graham and Y.Fregnac. Voltage-clamp measurement of visually-evoked conductances with whole-cell patch recordings in primary visual cortex. *J. Physiol Paris*, 90:185–188, 1996.

[46] L. Borg-Graham, C. Monier, and Y. Fregnac. Visual input evokes transient and strong shunting inhibition in visual cortical neurons. *Nature*, 393:369–373, 1998.

[47] D. Pare, E. Shink, H. Gaudreau, A. Destexhe, and E.J. Lang. Impact of spontaneous synaptic activity on the resting properties of cat neocortical pyramidal neurons in vivo. *J Neurophysiol*, 79:1450–1460, 1998.

[48] M.N. Shadlen and W.T. Newsome. The variable discharge of cortical neurons: implications for connectivity, computation and information coding. *J Neurosci*, 18:3870–3896, 1998.

[49] I. Lampl, I. Reichova, and D. Ferster. Synchronous membrane potential fluctuations in neurons of the cat visual cortex. *Neuron*, 22:361–374, 1999.

[50] J. Anderson, I. Lampl, D. Gillespie, and D. Ferster. The contribution of noise to contrast invariance of orientation tuning in cat visual cortex. *Science*, 290:1968–1972, 2000.

[51] M Shelley, D. McLaughlin, R. Shapley, and J. Wielaard. States of high conductance in a large-scale model of the visual cortex. *J. Comput. Neurosci.*, 13:93–109, 2002.

[52] A. Destexhe, M. Rudolph, and D. Pare. The high-conductance state of neocortical nurons in vivo. *Nat. Rev., Neurosci*, 4:730–751, 2003.

[53] D.A. Leopold and N.K. Logothetis. Spatial patterns of spontaneous local field activity in the monkey visual cortex. *Rev. Neurosci.*, 14:195–205, 2003.

[54] D.A. Leopold, Y. Murayama, and N.K. Logothetis. Very slow activity fluctuations in monkey visual cortex: implications for functional brain imaging. *Cereb. Cortex*, 13:422–33, 2003.

[55] D. McLaughlin, M. Shelley, R. Shapley, and J. Jin. High conductance dynamics of the primary visual cortex.

[56] D. Cai, L. Tao, M. Shelley, and D.W. Mclaughlin. An effective representation of fluctuation-driven neuronal networks with application to simple & complex cells in visual cortex. *Pro. Nat. Acad. Sci. (USA)*, 101:7757–7762, 2004.

[57] Y. Ikegaya, G. Aaron, R. Cossart, D. Aronov, I. Lampl, D. Ferster, and R. Yuste. Synfire chains and cortical songs: Temporal modules of cortical activity. *Science*, 304:559–564, 2004.

[58] L. Tao, D. Cai, D. McLaughlin, M.J. Shelley, and R. Shapley. Orientation selectivity in visual cortex by fluctuation-controlled criticality. *Proc. Natl. Acad. Sci.*, 103:102911–12916, 2006.

[59] A. Mokeichev, M. Okun, O. Barak, Y. Katz, O. Ben-Shahar, and I. Lampl. Stochastic emergence of repeating cortical motifs in spontaneous membrane potential fluctuations in vivo. *Neuron*, 53:319–21, 2007.

[60] L.G. Nowak, M.V. Sanchez-Vives, and D.A. McCormick. Spatial and temporal features of synaptic to discharge receptive field transformation in cat area 17. *J.Neurophysiol.*, 103:677–697, 2009.

[61] K. Fox and H. Sato and. N. Daw. The location and function of NMDA receptors in cat and kitten visual cortex. *J. Neurosci.*, 9:2443–2454, 1998.

[62] A.M. Rosier, L. Arckens, G.A. Orban, and F. Vandesande. Laminar distribution of nmda receptors in cat and monkey visual cortex visualized by [3h]-mk-801 binding. *J. Comp. Neurol.*, 335:369–380, 1993.

[63] N.W. Daw, P.G.S. Stein, and K. Fox. The role of nmda receptors in information transmission. *Annu Rev Neurosci*, 16:207–222, 1993.

[64] G.W. Huntley, J.C. Vickers, N. Brose, S.F. Heinemann, and J.H. Morrison. Distribution and synaptic localization of immunocytochemically identified nmda receptor subunit proteins in sensory motor and visual cortices of monkey and human. *J Neurosci.*, 14:3603–3619, 1994.

[65] C.E. Schroeder, D.C. Javitt, M. Steinschneider, A.D. Mehta, S.J. Givre, H.G. Vaughan, and Jr. J.C. Arezzo. N-methyl-d-aspartate enhancement of phasic responses in primate neocortex. *Exp Brain Res*, 114:271–278, 1997.

[66] D. Feldmeyer, V. Egger, J. Lubke, and B. Sakmann. Reliable synaptic connections between pairs of excitatory layer 4 neurones within a single barrel of developing rat somatosensory cortex. *Journal of Physiology*, 521:169–190, 1999.

[67] X.J. Wang. Synaptic basis of cortical persistent activity: the importance of nmda receptors to working memory. *J Neurosci.*, 19:9587–9603, 1999.

[68] A.J. Watt, M.C.W. Rossum, K.M. MacLeod, S.B. Nelson, and G.G. Turrigiano. Activity coregulates quantal ampa and nmda currents at neocortical synapses. *Neuron*, 26:659–670, 2000.

[69] C. Rivadulla, J. Sharma, and M. Sur. Specific roles of nmda and ampa receptors in direction-selective and spatial phase-selective responses in visual cortex. *J. Neurosci.*, 21:1710–1719, 2001.

[70] C. Myme, K. Sugino, G. Turrigiano, and S.B. Nelson. The nmda-to-ampa ratio at synapses onto layer 2/3 pyramidal neurons is conserved across prefrontal and visual cortices. *J Neurophysiol*, 90:771–779, 2003.

[71] P. Dayan and L.F. Abbott. *Theoretical Neuroscience.* MIT press, Cambridge, MA, 2001.

[72] J.S. Lund. Anatomical organization of macaque monkey striate visual cortex. *Ann. Rev. Neurosci.*, 11:253–288, 1988.

[73] R. De Valois, D. Albrecht, and L. Thorell. Spatial frequency selectivity of cells in macaque visual cortex. *Vision Res*, 22:545–559, 1982.

[74] R. Everson, A. Prashanth, M. Gabbay, B. Knight, L. Sirovich, and E. Kaplan. Representation of spatial frequency and orientation in the visual cortex. *Proc. Natl. Acad. Sci. USA*, 95:8334–8338, 1998.

[75] L. Tao, M. Shelley, D. McLaughlin, and R. Shapley. An egalitarian network model for the emergence of simple and complex cells in visual cortex. *Proc Natl Acad Sci U.S.A.*, 101(1):366–371, 2004.

[76] T. Bonhoeffer and A. Grinvald. Iso-orientation domains in cat visual cortex are arranged in pinwheel like patterns. *Nature*, 353:429–431, 1991.

[77] Y. Li and S. Wiggins. *Invariant Manifolds and Their Fibrations for Perturbed NLS Pdes Graph Transform Approach.* Springer-Verlag (New York), 1997.

[78] C. Beaulieu, Z. Kisvarday, P. Somogyi, M. Cynader, and A. Cowey. Quantitative distribution of GABA immunopositive and immunonegative neurons and synapses in the monkey striate cortex. *Cerebral Cortex*, 2:295–309, 1992.

[79] C. Rao, L.J. Toth, and M. Sur. Optically imaged maps of orientation preference in primary visual cortex of cats and ferrets. *J. Comp. Neurology*, 387:358–370, 1997.

[80] B. Pakkenberg and H.J. Gundersen. Neocortical neuron number in humans: effect of sex and age. *J. Comp. Neurol.*, 384:312–20, 1997.

[81] I. Levy, U. Hasson, and R. Malach. One picture is worth at least a million neurons. *Curr. Bio.*, 14:996–1001, 2004.

[82] J. DeFelipe and A. Fairen. Synaptic connections of an interneuron with axonal arcades in the cat visual cortex. *J. Neurocytol.*, 17:313–23, 1988.

[83] N.H. Yabuta and E.M. Callaway. Functional streams and local connections of layer 4C neurons in primary visual cortex of the macaque monkey. *J. Neurosci.*, 18:9489–499, 1998.

[84] E.M. Callaway. Cell type specificity of local cortical connections. *J. Neurocyt.*, 31:231–37, 2002.

[85] G. Buzsaki, C. Geisler, D.A. Henze, and X.J. Wang. Interneuron diversity series: Circuit complexity and axon wiring economy of cortical interneurons. *Trends Neurosci.*, 27:186–93, 2004.

[86] A. Mason, A. Nicoll, and K. Stratford. Synaptic transmission between individual pyramidal neurons of the rat visual cortex in vitro. *J. Neurosci*, 11:72–84, 1991.

[87] K. Tarczy-Hornoch, K.A. Martin, J.J. Jack, and K.J. Stratford. Synaptic interactions between smooth and spiny neurones in layer 4 of cat visual cortex in vitro. *J. Physiol*, 508:351–63, 1998.

[88] H. Sato, Y. Hata, and T. Tsumoto. Effects of blocking non-n-methyl-d-aspartate receptors on visual responses of neurons in the cat visual cortex. *Neuroscience*, 94:697–703, 1999.

[89] D. Ferster, S. Chung, and H. Wheat. Orientation selectivity of thalamic input to simple cells of cat visual cortex. *Nature*, 380:249–252, 1996.

[90] J.M. Ichida, L. Schwabe, P.C. Bressloff, and A. Angelucci. Response facilitation from the 'suppressive' receptive field surround of macaque v1 neurons. *J. Neurophysiol.*, 98:2168–2181, 2007.

[91] G. Blasdel. Orientation selectivity, preference, and continuity in the monkey striate cortex. *J. of Neurosci.*, 12:3139–3161, 1992.

[92] J. Crook, Z. Kisvarday, and U. Eysel. Gaba induced inactivation of functionally characterized sites in cat striate cortex: Effects on orientation tuning and direction selectivity. *Nature*, 14:141–158, 1997.

[93] M.A. Gieselmann and A. Thiele. Comparison of spatial integration and surround suppression characteristics in spiking activity and the local field potential in macaque v1. *European J.Neurosci.*, 28:447–459, 2008.

[94] G. Kovacic, A.V. Rangan, L. Tao, , and D. Cai. Fokker-planck description of conductance-based integrate-and-fire neuronal networks. *Phys. Rev. E*, 80:021904, 2009.

[95] A.V. Rangan and L.S. Young. Network mechanisms involving collaborative activity within a model of the visual cortex. *Journal of Computational Neuroscience*, to be submitted, 2012.

[96] J.S. Anderson, M. Carandini, and D. Ferster. Orientation tuning of input conductance, excitation, and inhibition in cat primary visual cortex. *J. Neurophysiol.*, 84:909–926, 2000.

[97] G. Gonzalez-Burgos, G. Barrionuevo and D.A. Lewis. Horizontal Synaptic Connections in Monkey Prefrontal Cortex: An In Vitro Electrophysiological Study. *Cereb. Cortex.*, 10:82-92, 2000.

[98] P. Girard and J.M. Hupe and J. Bullier. Feedforward and feedback connections between areas V1 and V2 of the monkey have similar rapid conduction velocities. *J. Neurophysiol.*, 85:1328-1331, 2001.

[99] J.A. Hirsch, L.M. Martinez, C. Pillai, J.M. Alonso, Q.B. Wang, and F.T. Sommer. Functionally distinct inhibitory neurons at the first stage of visual cortical processing. *Nat. Neurosci.*, 6:1300–1308, 2003.

[100] N.J. Priebe and D. Ferster. Inhibition, spike threshold, and stimulus selectivity in primary visual cortex. *Neuron*, 57:482–97, 2008.

[101] B.H. Liu, P.Y. Li, Y.T. Li, Y.J.J. Sun, Y. Yanagawa, K. Obata, L.I. Zhang, and H.Z.W. Tao. Visual receptive field structure of cortical inhibitory neurons revealed by two-photon imaging guided recording. *J. Neurosci.*, 29:10520–32, 2009.

[102] T. Troyer, A. Krukowski, N. Priebe, and K. Miller. Contrast invariant orientation tuning in cat visual cortex with feedforward tuning and correlation based intracortical connectivity. *J. Neurosci.*, 18:5908–5927, 1998.

[103] Y. Banitt, K.A.C. Martin, and I. Segev. A biologically realistic model of contrast invariant orientation tuning by thalamocortical synaptic depression. *J. Neurosci.*, 19:10230–10239, 2007.

[104] J.P. Jones and L.A. Palmer. The two-dimensional spatial structure of simple receptive fields in cat striate cortex. *J Neurophysiol. 58:1187-211*, 1987.

[105] D. Ringach. Spatial structure and symmetry of simple-cell receptive fields in macaque primary visual cortex. *J. Neurophysiol.*, 88:455–463, 2002.

[106] A. Angelucci, J.B. Levitt, P. Adorjan, Y. Zheng, L.C. Sincich, N.P. McLoughlin, G.P. Blasdel, and J.S. Lund. Bar-like patterns of lateral connectivity in layers 4B and upper 4Cα of macaque primary visual cortex, area V1. preprint.

[107] D.L. Ringach, M.J. Hawken, and R. Shapley. Dynamics of orientation tuning in macaque v1: The role of global and tuned suppression. *J.N. Physiol.*, 90:342–50, 2002.

[108] C. Chiu and M. Weliky. Spontaneous activity in developing ferret visual cortex in vivo. *J. Neurosci.*, 21:8906–8914, 2001.

[109] T. Kenet, D. Bibitchkov, M. Tsodyks, A. Grinvald, and A. Arieli. spontaneously emerging cortical representations of visual attributes. *Nature*, 425:954–956, 2003.

[110] J.A. Goldberg, U. Rokni, and H. Sompolinsky. Patterns of ongoing activity and the functional architecture of the primary visual cortex. *Neuron*, 13:489–500, 2004.

[111] D. Cai, A.V. Rangan, and D. McLaughlin. Architectural and synaptic mechanisms underlying coherent spontaneous activity in v1. *Proc. Natl. Acad. Sci. (USA)*, 102:5868, 2005.

[112] B.K. Murphy and K.D. Miller. Balanced amplification: A new mechanism of selective amplification of neural activity patterns. *Neuron*, 61:635–648, 2009.

[113] B. Blumenfeld, D. Bibitchkov, and M. Tsodyks. Neural network model of the primary visual cortex: From functional architecture to lateral connectivity and back. *J. Comput. Neurosci.*, 20:219–241, 2006.

[114] I. Ayzenshtat, E. Meirovithz, H. Edelman, U. Werner-Reiss, E. Bienenstock, M. Abeles, and H. Slovin. Precise spatiotemporal patterns among visual cortical areas and their relation to visual stimulus processing. *J. Neurosci.*, 18:11232–11245, 2010.

[115] A. Mazzoni, F.D. Broccard, E. Garcia-Perez, P. Bonifazi, M.E. Ruaro, and V. Torre. On the dynamics of the spontaneous activity in neuronal networks. *PLoS One*, 5:e439, 2007.

[116] Mark M Churchland, Byron M Yu, John P Cunningham, Leo P Sugrue, Marlene R Cohen, Greg S Corrado, William T Newsome, Andrew M Clark, Paymon Hosseini, Benjamin B Scott, David C Bradley, Matthew A Smith, Adam Kohn, J Anthony Movshon, Katherine M Armstrong, Tirin Moore, Steve W Chang, Lawrence H Snyder, Stephen G Lisberger, Nicholas J Priebe, Ian M Finn, David Ferster, Stephen I Ryu, Gopal Santhanam, Maneesh Sahani, and Krishna V Shenoy. Stimulus onset quenches neural variability: a widespread cortical phenomenon. *Nature Neuroscience*, 13:369–378, 2010.

[117] G.C. DeAngelis, R.D. Freeman, and I. Ohzawa. Length and width tuning of neurons in the cat's primary visual cortex. *J. Neurophys.*, 71:347–74, 1994.

[118] H.E. Jones, W. Wang, and A.M. Sillito. Spatial organization and magnitude of orientation contrast interactions in primate v1. *J. Neurophys.*, 88:2796–808, 2002.

[119] L. Schwabe, J.M. Ichida, S. Shushruth, P. Mangapathy, and A. Angelucci. Contrast-dependence of surround suppression in macaque v1: Experimental testing of a recurrent network model. *Neuroimage*, 52:777–792, 2010.

[120] X.M. Song and C.Y. Li. Contrast-dependent and contrast-independent spatial summation of primary visual cortical neurons of the cat. *Cereb. Cortex*, 18:331–336, 2008.

[121] R. Douglas, C. Koch, M. Mahowald, K. Martin, and H. Suarez. Recurrent excitation in neocortical circuits. *Science*, 269:981–985, 1995.

[122] H.S. Seung. *The Handbook of Brain Theory and Neural Networks.* Cambridge, MA: MIT Press, 2003.

[123] R. Eckhorn, R. Bauer, W. Jordan, M. Brosch, W. Kruse, M. Munk, and H.J. Reitboeck HJ. Coherent oscillations: a mechanism of feature linking in the visual cortex? multiple electrode and correlation analyses in the cat. *Biol. Cybern*, 60:121–130, 1988.

[124] W. Singer and C.M. Gray. Visual feature integration and the temporal correlation hypothesis. *Annu Rev Neurosci.*, 18:555–586, 1995.

[125] C. van Vreeswijk and H. Sompolinsky. Chaos in neuronal networks with balanced excitatory and inhibitory activity. *Science*, 274:1724–1726, 1996.

[126] E.T. Rolls, A. Treves, and M.J. Tovee. The representational capacity of the distributed encoding of information provided by populations of neurons in primate temporal visual cortex. *Exp. Brain Res.*, 114:149–162, 1997.

[127] E.T. Rolls, A. Treves, M.J. Tovee, and S. Panzeri. Information in the neuronal representation of individual stimuli in the primate temporal visual cortex. *J. Comp. Neurosci*, 4:309–333, 1997.

[128] T.P. Vogels and L.F. Abbott. Gating multiple signals through detailed balance of excitation and inhibition in spiking networks. *Nat. Neurosci.*, 12:483–91, 2009.

[129] K. Miura, Y. Tsubo, M. Okada, and T. Fukai. Balanced excitatory and inhibitory inputs to cortical neurons decouple firing irregularity from rate modulations. *J. Neurosci.*, 27:13802–12, 2007.

[130] R.W. Berg and J. Hounsgaard. Signaling in large-scale neural networks. *Cogn. Process.*, 10:S9–15, 2009.

[131] S. Shew, H. Yang, S. Yu, R. Roy, and D. Plenz. Information capacity and transmission are maximized in balanced cortical networks with neuronal avalanches. *J. Neuroscience*, 31:55–63, 2011.

[132] N. Brunel and V. Hakim. Fast global oscillations in networks of integrate-and-fire neurons with low firing rates. *Neural Comp.*, 11:1621–1671, 1999.

[133] N. Brunel. Dynamics of sparsely connected networks of excitatory and inhibitory spiking neurons. *J. Comp. Neurosci.*, 8:183–208, 2000.

[134] J.R. Mller and A.B. Metha and J. Krauskopf and P. Lennie. Rapid adaptation in visual cortex to the structure of images. *Science*, 285, 1405-1408, 1999.

[135] N. Fourcaud and N. Brunel. Dynamics of the firing probability of noisy integrate-and-fire neurons. *Neural Comp.*, 14:2057–2110, 2002.

[136] C. Geisler, N. Brunel, and X.J. Wang. Contributions of intrinsic membrane dynamics to fast network oscillations with irregular neuronal discharges., 2005.

[137] S. Cardanobile and S. Rotter. Multiplicatively interacting point processes and applications to neural modeling. *J. Comp. Neurosci.*, 28:267–284, 2010.

[138] D. Hansel and H. Sompolinsky. Chaos and synchrony in a model of a hypercolumn in visual cortex. *J. Comp. Neurosci.*, 3:7–34, 1996.

[139] D. Zhou, Y. Sun, A.V. Rangan, , and D. Cai. Network-induced chaos in integrate-and-fire neuronal ensembles. *Phys. Rev. E*, 80:031918, 2008.

[140] S. Yu, H. Yang, H. Nakahara, G.S. Santos, D. Nikolic, and D. Plenz. Higher-order interactions characterized in cortical activity. *J. Neurosci.*, 31:17514–17526, 2011.

[141] A.S. Ecker, P. Berens, G.A. Keleris, M. Bethge, N.K. Logothetis, and A.S. Tolias. Decorrelated neuronal firing in cortical microcircuits. *Science*, 29:584–587, 2010.

[142] W. Henrie and R. Shapley. LFP power spectra in V1 cortex: the graded effect of stimulus contrast. *J. Neurophysiol.*, 94:479-90, 2005.

[143] U. Mitzdorf. Properties of the evoked potential generators: current source density analysis of visually evoked potentials in the cat cortex. *Int. J. Neurosci.*, 33:33–59, 1987.

[144] S. Katzner, I. Nauhaus, A. Benucci, V. Bonin, D.L. Ringach, and M. Carandini. Local origin of field potentials in visual cortex. *Neuron*, 61:35–41, 2009.

[145] J. Yu and D. Ferster. Membrane potential synchrony in primary visual cortex during sensory stimulation *Neuron*, 68:1187–1201, 2010.
